# The theory of epidemics with altruism

**DOI:** 10.1073/pnas.2518893123

**Published:** 2026-02-27

**Authors:** Mark P. Lynch, Simon K. Schnyder, John J. Molina, Ryoichi Yamamoto, Matthew S. Turner

**Affiliations:** ^a^Department of Mathematics, Mathematics for Real-World Systems Centre for Doctoral Training, University of Warwick, Coventry CV4 7AL, United Kingdom; ^b^Institute of Industrial Science, The University of Tokyo, Tokyo 153-8505, Japan; ^c^Department of Chemical Engineering, Kyoto University, Kyoto 615-8510, Japan; ^d^Department of Physics, University of Warwick, Coventry CV4 7AL, United Kingdom; ^e^Institute for Global Pandemic Planning, University of Warwick, Coventry CV4 7AL, United Kingdom

**Keywords:** epidemiology, game theory, control theory, mean-field games, mathematical modeling

## Abstract

Social distancing can mitigate the spread of diseases in humans and animals. Social distancing allows susceptible individuals to protect themselves (and others) but confers no personal benefit for infected individuals if recovery provides immunity. However, individuals are likely to be at least weakly altruistic and may be interested in protecting others when infected. A strongly altruistic population where individuals value others as equal to themselves would be expected to self-isolate when infected. This would strongly suppress the disease, avoid Herd Immunity, and vastly improve outcomes. Still, little is known about how weaker altruism affects behavior during epidemics. Here we show using game theory that even extremely weakly altruistic individuals, valuing their own lives equivalent to roughly 100,000 others, can rationally achieve almost identical outcomes. Individuals self-isolate in order to avoid setting off chains of infections that they would perceive as costly to them even at such small altruism. Our results are robust to a moderate fraction of asymptomatic cases or completely selfish individuals. The resulting behavior, while emerging from a complex optimization problem, is simple enough that it could have evolved as a behavioral response in social animals, as well as being easy to communicate and understand for humans.

## Significance

The question of whether to social distance when infected with a dangerous disease can be viewed as a problem in game theory. We find that such behavior is only rational for individuals with a minimum level of altruism, quantifying how much they care about the outcomes of others in the population. Remarkably, the altruism threshold above which it is rational to protect others like this can be extremely low: valuing one’s own life as equivalent to 100,000 others. Our work may be useful for policy makers. It may also help understand the behavior of animals living in groups of related kin. Here, a similar Nash equilibrium exists in which it would be advantageous for animals to socially distance when sick.

Epidemics of infectious diseases occur frequently in human and animal societies. When faced with a sufficiently dangerous disease, individuals can react by modifying their behavior in order to mitigate their chances of infection (1–10). To understand, predict, and potentially guide epidemic outcomes, it is essential to model not only disease transmission (11) but also the goals and motivations behind behavioral responses (12–22).

Two extreme cases are well understood: the socially optimal behavior that maximizes a population objective function and self-interested behavior yielding Nash equilibrium solutions, where no individual can deviate to improve their private objective function. An often neglected motivation is altruism: social individuals being invested in the fate of others. Despite its importance, it is still poorly understood how partial altruism would manifest in self-organized behavior during an epidemic (23). The effect of social distancing depends on the state of the individual: It allows susceptible individuals to protect themselves (and others) but confers no personal benefit for infected individuals, although they are still able to protect others.

Our approach meets key criteria: It employs decentralized decision-making, accounts for different behaviors based on infection status, uses interpretable cost-based objective functions, and leads to stable Nash equilibria. Importantly, our approach allows us to take into account the full time course of an epidemic. Previous studies have modeled altruism in differing ways. While they are successful in certain aspects, none meet all of our criteria for altruism. The most relevant are: Quaas et al. (24) analyze behavior generated by combining the purely selfish and utilitarian behaviors. This approach only yields Nash equilibria at the trivial extremes. Brotherhood et al. (25) only model altruistic behavior via the indirect proxy of individuals having a preference for staying at home, rather than directly accounting for the costs of others. Alfaro et al. (26) use a model in which perfect altruism does not line up with the social optimum. Toxvaerd (27) uses partial altruism of two agents when deciding if they want to interact given respective infection probabilities, but does not investigate the impact on a population scale epidemic. The influential work of Farboodi et al. (22) is restricted to the special case of perfectly asymptomatic infection in which there is no possibility of infected individuals behaving differently, a key feature of the present work; this would have had a significant effect on results involving partial altruism but that case was not explicitly studied in their work.

Here, we show that remarkably low levels of altruism can lead to a Nash equilibrium that strongly suppresses a disease, even when a significant fraction of cases remains asymptomatic (28–31). Behavior can be highly sensitive to the strength of altruism, leading to outcomes that are close to the worst, and best, possible. Understanding this is the primary objective of this work. We hope these results can help motivate self-isolation by infected individuals by revealing its rationality. The improved understanding we provide could be invaluable in the design of policy interventions to leverage the effect of partial altruism.

In social animals altruism can be interpreted as a form of kin selection. Given how little altruism is required to motivate self-isolation by infected individuals, this behavior could be expected to arise via natural selection, even in quite distantly related groups of animals. While unambiguous evidence for this phenomenon are lacking, except perhaps in eusocial animals, some observations are suggestive, e.g., sick animals i) leaving a social group (to die) and ii) reducing their social signaling (1, 32–34). Our work may help to motivate further research in this area.

## Epidemic Dynamics with Behavioral Control

We propose a formulation of SIR dynamics (11) that differentiates between a representative individual and the rest of the population (simply referred to as the population in what follows) in a way that allows us to reconstruct the population as a collection of individuals. This represents a mean-field game (14, 18, 35–37), used in many behavioral epidemiological models (16–20, 38–43). Importantly, in our model, we carefully expose the infinitesimal effect of individuals on the course of the epidemic. Because of its small size, this effect seems to have been overlooked so far, but makes game theoretic analysis possible.

The state of the epidemic is given by the proportion of the population who are either susceptible, infected, or recovered from the disease s(t), i(t), and r(t), respectively. We consider the case where the populations in each compartment are allowed to choose different levels of social activity and write these as $k_{s}$, $k_{i}$, and $k_{r}$, respectively, with $k_{j}\in \left[0,1\right]$ for j∈{s,i,r}. Here, $k_{j}=1$ represents baseline population behavior in the absence of an epidemic, while $k_{j}=0$ is complete self-imposed isolation, noting that our results are qualitatively insensitive to a restriction that very small $k_{j}\ll 1$ are behaviorally inaccessible. To understand the decision-making it is necessary to differentiate the population state from the probability that an individual is in each state, written *p*_*s*_(*t*), *p*_*i*_(*t*) and *p*_*r*_(*t*) respectively. Individuals have a social activity, depending on their state, written $\kappa_{s}$, $\kappa_{i}$ and $\kappa_{r}$ respectively, to differentiate these from the corresponding population activities $k_{j}$ over which individuals have no direct control. These three time-dependent social activities $\kappa_{j}\left(t\right)$ are the only control variables for our problem. The individual chooses their activities $\kappa_{j}\left(t\right)$ so as to maximize their objective function given a population behavior $k_{j}\left(t\right)$. We assume that the rest of the population is composed of individuals that are identical in their interests to the representative individual, their decision-making is the same and hence the individual and the population will finally be made self consistent according to $k_{j}=\kappa_{j}$, *s* = *p*_*s*_, *i* = *p_i_*, and *r* = *p*_*r*_. The distinction between individual and population states and controls must be maintained at the outset in order to enable the game theoretic reasoning behind the choice of compartmental behaviors and resulting disease dynamics.

Our mean field model can be rigorously derived from a microscopic model in which *N* individuals interact with each other in a well-mixed, mean-field setting. We then focus on one representative individual who interacts with the other N−1 individuals representing the remainder of the population. Infections occur with a certain probability when susceptible and infected individuals make a social contact, moving individuals from the susceptible to the infected compartment. In a well-mixed population, the contact rate between compartments (and the individual) is then given by the product of the fraction in each compartment (and the probability of the individual being in that compartment), and the product of the social activity of each (44). Other choices of contact rates are possible. Because the individual is only one member of the population of size *N*, its contribution to the total number of contacts that the remainder of the population experiences must scale with 1/N, while the rest of the population makes up the rest of contacts, at a proportion of 1−1/N.

We assume that recovered individuals cannot be reinfected or spread the disease, thus we need not analyze them explicitly in what follows. We choose time units equal to the recovery time so that, in the absence of behavioral modification $k_{j}=\kappa_{j}=1$, infection occurs at a rate proportional to the basic reproduction number $R_{0}$ (3). The full dynamics are then written

[1] $$\begin{array}{cc} \frac{\mathrm{ds}}{\mathrm{dt}} & =-R_{0}k_{s}s\left(\left(1-\frac{1}{N}\right)k_{i}i+\frac{1}{N}\kappa_{i}p_{i}\right)\\ \frac{\mathrm{di}}{\mathrm{dt}} & =R_{0}k_{s}s\left(\left(1-\frac{1}{N}\right)k_{i}i+\frac{1}{N}\kappa_{i}p_{i}\right)-i\\ \frac{dp_{s}}{\mathrm{dt}} & =-R_{0}\kappa_{s}k_{i}p_{s}i\\ \frac{dp_{i}}{\mathrm{dt}} & =R_{0}\kappa_{s}k_{i}p_{s}i-p_{i} \end{array}$$

with boundary conditions $i\left(0\right)=p_{i}\left(0\right)=i_{0}$ and $s\left(0\right)=p_{s}\left(0\right)=1-i_{0}$. The time origin t=0 represents the start of the epidemic. A more complete derivation is given in *SI Appendix*, section 3.B.1.

### Decision-Making Process.

In order to incorporate partial altruism, we must define an objective function that includes the interest of the individual in the outcomes for other members of society. An intuitively accessible way of quantifying altruism is how much an individual cares about a randomly selected other individual as compared to themselves. This simple intuition can be formalized into the altruism parameter χ≥0 which represents the relative importance assigned to the outcomes of others. For instance, an altruism of χ=1 then means that an individual cares about every individual in the population—including themselves—equally.

We define an objective function *U* that depends on the individual’s infection state and behavior, as well as the state and behavior of the population. We incorporate population costs into the objective function $U=\int_{0}^{t_{f}}u\;dt+U_{f}$ by defining the cost per unit time

[2] $$\begin{array}{cc} u & =-\chi \left(N-1\right)(\alpha i+\omega_{s}s{\left(k_{s}-1\right)}^{2}+\omega_{i}i{\left(k_{i}-1\right)}^{2})\\ & -(\alpha p_{i}+\omega_{s}p_{s}{\left(\kappa_{s}-1\right)}^{2}+\omega_{i}p_{i}{\left(\kappa_{i}-1\right)}^{2}) \end{array}$$

Here, *α* is the cost associated with contracting the disease and $\omega_{s}$, $\omega_{i}$ parameterize the costs of social distancing for those who are susceptible or infected, respectively. While we use a social distancing cost of simple quadratic form (19, 20) one could use other functional forms that have a nonlinear control dependence (45), such as a logarithmic cost (22). In what follows, we simplify by assuming further that $\omega_{i}=\omega_{s}=\omega$ and then choose units for the objective function in which ω=1 without further loss of generality.

At χ=0, the individual is acting completely in its own self interest while for χ=1 the individual coequates their own costs with those of each of the N−1 other members of the population. Thus an intuitive interpretation of 1/χ is that it is the number of population members that the individual would consider as coequal to themselves, if both suffered the same costs. In our formulation, there is no need to restrict χ≤1. One could imagine a population of hyperaltruistic individuals, χ>1. This is not the main focus of the present work but we will briefly touch on this case in the context of asymptomatic infections.

The term $U_{f}$ encodes costs that are incurred after some final time $t_{f}$. In this work, we assume that at $t_{f}$ the susceptible population becomes immune, e.g via a perfect vaccination, and $U_{f}=-\alpha \left(p_{i}\left(t_{f}\right)+\chi \left(N-1\right)i\left(t_{f}\right)\right)$ (See *SI Appendix*, section 2.C.2 for details). For animals, it might be reasonable to assume that $t_{f}\to \infty$.

A Nash equilibrium is reached when no individual can find an alternative strategy that further improves their outcome. This would then correspond to the whole population adopting the same behavior. Such solutions are formally obtained by maximizing *U* with respect to the $\kappa_{j}$ and then setting $k_{j}=\kappa_{j}$. As a result, the population and individual dynamics also align perfectly, *s* = *p*_*s*_, etc.

Only after deriving the Nash equilibrium behavior, we perform the limit in which the population becomes infinitely large, N→∞. In this limit, the effect of the individual on the course of the epidemic in the infinitely large population becomes infinitely small, scaling with 1/N, Eq. **1**, but the value of the population costs in the utility, scaling with *N*, Eq. **2**, becomes infinitely large. Importantly, these two effects balance each other out in the equations determining the behavior of the individual: the contribution to the utility that is under the control of the individual remains finite. If this limit were taken immediately after writing Eq. **1**, the effect of the individual on the population dynamics would vanish, trivially decoupling the individual and population dynamics. Full details are given in *Materials and Methods*.

A Nash equilibrium, however, does not necessarily correspond to the best possible outcome accessible to the population. This is given by the utilitarian maximum, defined as the behavior $k_{j}$ adopted by everyone in the population that leads to the maximal possible value of the objective function. Formally, this is found by setting $k_{j}=\kappa_{j}$ before maximizing the objective function over $k_{j}$. This requires complete control over the population. This could be realized by a benevolent “social planner” who can control the behavior of the whole population and target this solution. In eusocial animals and clonal populations, this control could arise via natural selection. Discussion of this traditional utilitarian maximum is left for *SI Appendix*, section 5.B (and Self-Interested in *SI Appendix*, section 5.A). Reassuringly, within our model, this case identically corresponds to χ=1 in which each individual values the costs to any member of the population the same as to themselves. In this case the nash equilibrium returns the maximal value of the objective function, thus creating a utilitarian equilibrium (*SI Appendix*, section 3.B.4).

## Results

### Sufficiently Altruistic Populations Can Spontaneously Suppress Diseases.

**Fig. 1.**
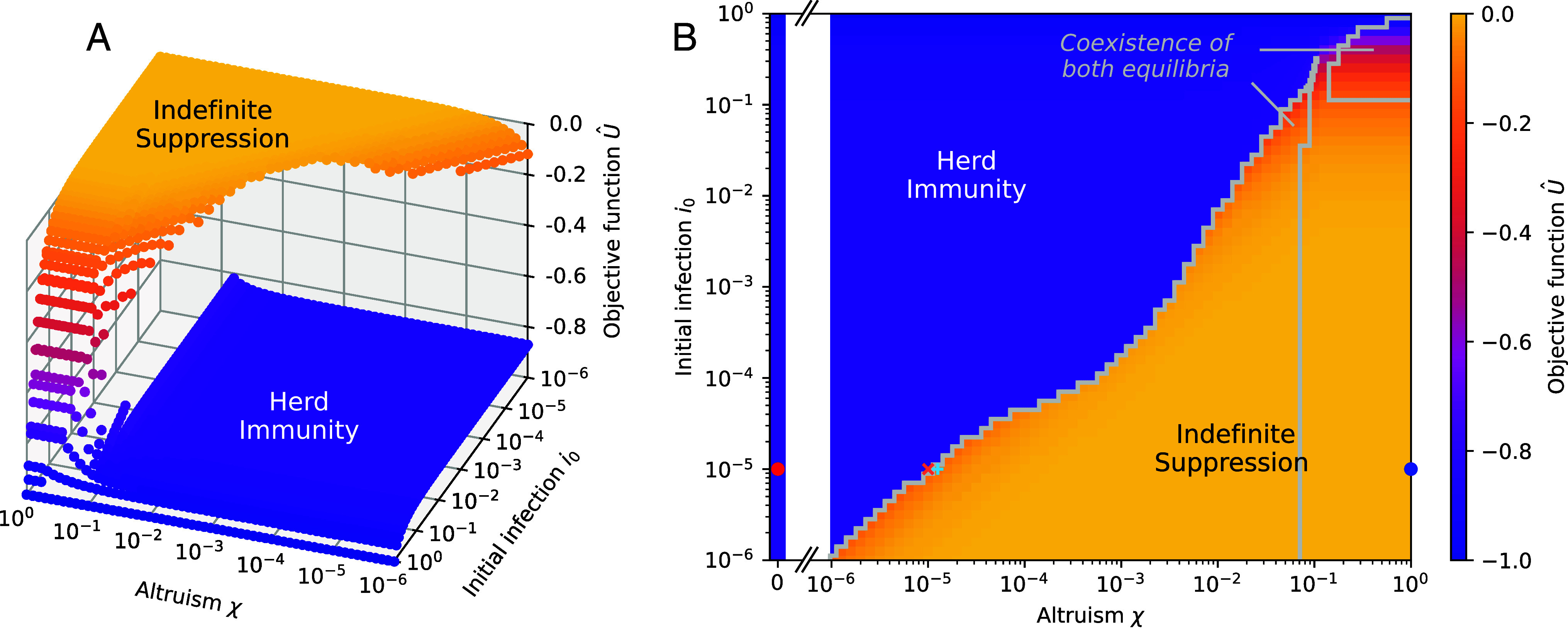
The disease dynamics is characterized by two Nash equilibria with vastly different outcomes. (*A*) The scaled value of the objective function within the individual’s frame of reference $\hat{U}=\;\lim_{N\to \infty }U/\alpha \left(1+\chi \left(N-1\right)\right)$ realized for the two Nash equilibria that emerge is shown as a function of initial infection numbers $i_{0}$ and altruism *χ*. Indefinite Suppression is characterized by strong social distancing of infected and negligible social distancing of susceptibles, while Herd Immunity is characterized by social distancing of susceptibles only. (*B*) The highest value of $\hat{U}$ for either Nash equilibrium. Symbols represent the parameter values for which the full system dynamics are shown in Fig. 2. The coexistence regions surrounded by gray lines show where there are two equilibria with scaled objective functions differing by more than $10^{-2}$. The resolution of the plot around the feature at the top of the main coexistence region at $\chi \approx 10^{-1}$ and $i_{0}\approx 0.3$ was increased in order to resolve it more accurately. Here, we show results for $\alpha =5,R_{0}=3,$ and $t_{f}=100$.

We find two Nash equilibria with very different outcomes, see Fig. 1. In the first equilibrium, Indefinite Suppression, the population acts to contain the disease throughout the epidemic. This results in a high value of the objective function (low cost). The other equilibrium, Herd Immunity, involves the disease infecting a large fraction of the population and has a low value of the objective function (high cost).

Both equilibria coexist over much of the parameter space, implying that a population might choose either, see Fig. 1, specifically the areas surrounded by gray in panel (*B*). In the majority of parameter space, given the vastly better outcome for the Indefinite Suppression equilibrium, it is clearly objectively preferable (for details on the coexistence region at large $i_{0}$ and *χ*, likely to be epidemiologically irrelevant, see *SI Appendix*, section 5.C).

It is remarkable that we find the Indefinite Suppression equilibrium persisting to extremely low values of altruism. We find that the altruism threshold at which the Indefinite Suppression equilibrium is lost, $\chi_{c}$, depends on the initial level of infections $i_{0}$. For example, with $i_{0}=10^{-5}$, corresponding to individuals becoming aware of the disease when there are a few hundred cases in a medium sized country, the threshold can be as small as $\chi_{c}=10^{-5}$, see Fig. 1*B*. This is an extraordinarily small number, corresponding to an individual valuing their own life as equivalent to the lives of $10^{5}$ others. The surprising implication of this is that extremely weak altruism can be sufficient to support the Indefinite Suppression equilibrium, with the enormous benefits this bestows. Qualitatively, the objective function landscape is remarkably insensitive to the precise choice of the remaining parameters *α*, $R_{0}$, and $t_{f}$ (*SI Appendix*, section 5.D and Figs. S5 and S6).

The Herd Immunity equilibrium is found to disappear for large altruism values $\chi ≳7\times 10^{-2}$, see Fig. 1*B*. This implies that a sufficiently altruistic population would target only an Indefinite Suppression equilibrium.

**Fig. 2.**
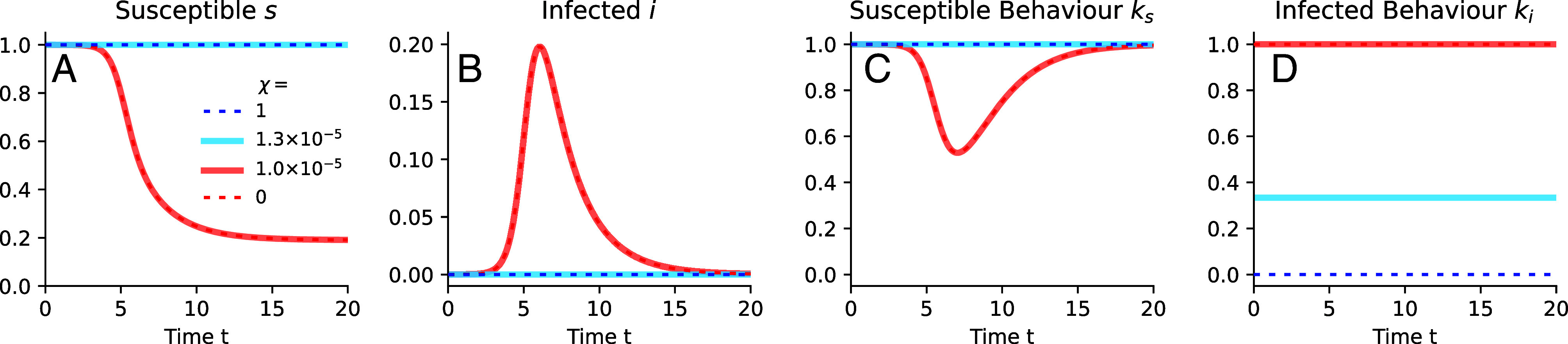
The disease dynamics are largely determined by the behavior of the infectious. The dynamics of the susceptible (*A*) and the infectious compartments (*B*), as well as the corresponding behaviors (*C* and *D*) are shown for initial infection level $i_{0}=10^{-5}$ for representative values of altruism *χ* on either side of the boundary of the Indefinite Suppression equilibrium at $\chi =\chi_{c}$, see corresponding colored points in Fig. 1*B*. Here, we show results for $\alpha =5,R_{0}=3,$ and $t_{f}=100$.

In order to characterize the two Nash equilibria, we examine representative disease dynamics, and the corresponding social behaviors, on either side of the boundary of Indefinite Suppression, see Fig. 2. The behaviors within each equilibrium are qualitatively similar, largely independent of $i_{0}$ and *χ*, but are very different between the two equilibria. Within the Indefinite Suppression equilibrium (blue lines), the infectious perform strong social distancing for most of the duration of the epidemic with the goal of protecting others, thus suppressing the disease strongly. As a result, the susceptible fraction of the population does not modify its behavior. Conversely, within the Herd Immunity equilibrium (red lines), altruism is weak enough such that the infectious largely do not perform social distancing (except at high $i_{0}$). The susceptible compartment responds with moderate social distancing in order to reduce their own probability of becoming infected. This strategy cannot avoid Herd Immunity but reduces the total number of cases as compared to the situation without behavioral modification. Hence, the infectious behavior is the key to understanding where these equilibria occur.

**Fig. 3.**
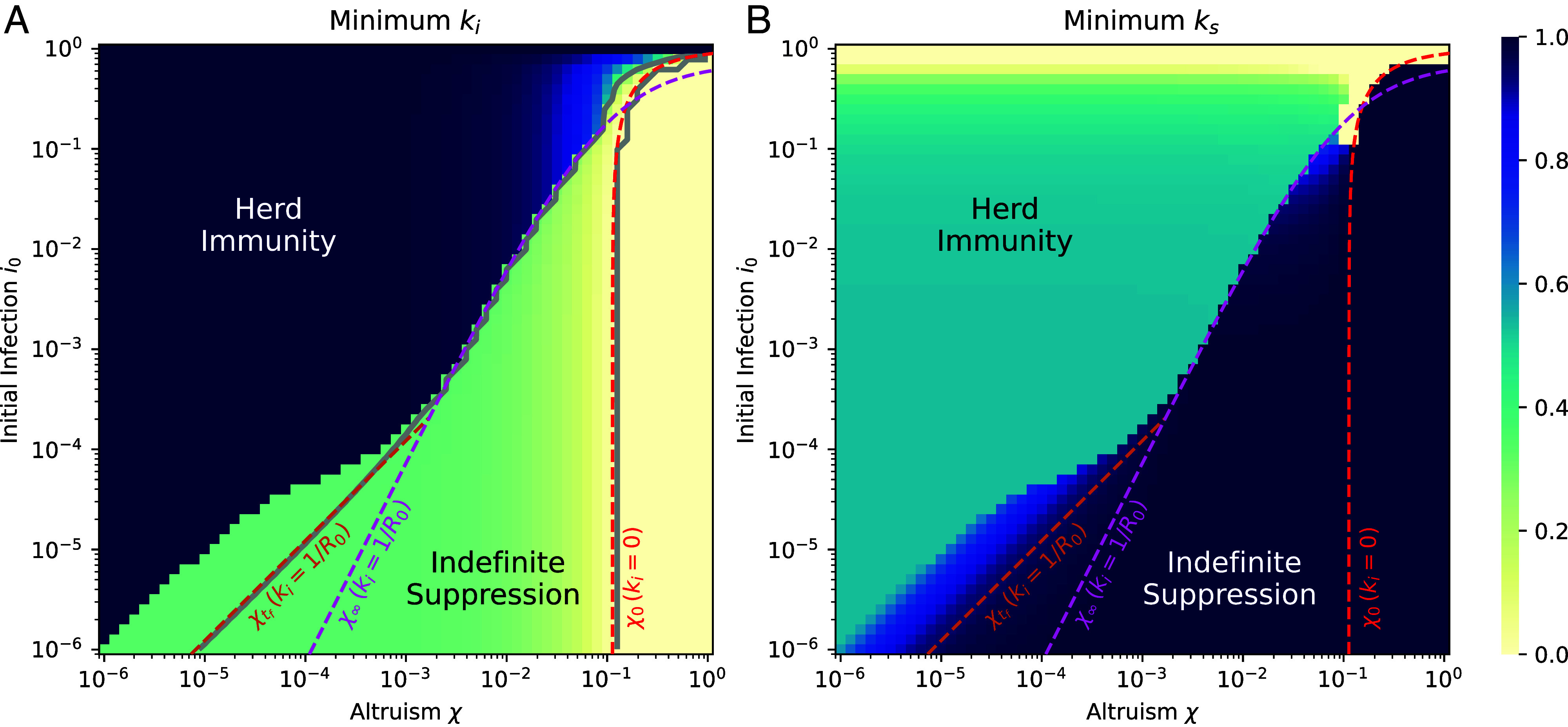
Minimum social activities characterize the equilibria: Marginal social distancing by infectious defines the edge of the Indefinite Suppression equilibrium. (*A*) Minimum of the infectious behavior and (*B*) the minimum of the susceptible behavior, for the Nash equilibria of best outcome. The gray solid lines in panel (*A*) represent numerical contours for constant $k_{i}$ values, $k_{i}=0$, and $k_{i}=1/R_{0}$. The red asymptote, $\chi_{0}$, encloses the region where the behavior is fully Utilitarian, $k_{i}=0$, and is in agreement with the corresponding numerical contour line, see Eq. **3**. The magenta asymptote is an analytic approximation to the boundary between the two Nash equilibria assuming an infinite time horizon $t_{f}\to \infty$, see Eq. **4**. It corresponds to $k_{i}=1/R_{0}$ and is in agreement with the corresponding numerical contour line for intermediate values of $i_{0}$. The orange asymptote describes the contour when a temporally constant behavior of $k_{i}=1/R_{0}$ and a finite time horizon $t_{f}=100$ are assumed, see Eq. **5**. This asymptote is in agreement with the numerical contour line for $k_{i}=1/R_{0}$ for small $i_{0}$. Here, we show results for $\alpha =5,R_{0}=3,$ and $t_{f}=100$.

For both equilibria, the behavior $k_{i}$ is almost precisely constant except for a late time transient, in which $k_{i}$ rapidly increases for *t* near $t_{f}$ (*SI Appendix*, Fig. S3). Exploiting this fact, we can examine $\min(k_{i})$ across the whole parameter space without losing deeper understanding, see Fig. 3*A*. In the Indefinite Suppression regime, the lower the value of *χ*, the weaker will be the incentive for infectious individuals to protect others from infection. When this incentive becomes too weak, the infected elect instead to no longer socially distance and Indefinite Suppression is lost. Indeed this is exactly what we find, as infectious behavior varies continuously from $k_{i}=0$ at χ=1 to $k_{i}\approx 1/R_{0}$ at the boundary $\chi =\chi_{c}$, while typically $k_{s}\approx 1$. We note that $k_{i}\leq 1/R_{0}$ is sufficient by itself to ensure that di/dt≤0 for all times. This is a key insight that allows us to construct analytical approximations describing lines of constant infectious behavior with $k_{s}=1$.

The Utilitarian maximum is realized when the infectious select $k_{i}=0$, corresponding to the fastest possible disease suppression. We predict this would occur for $\chi \geq \chi_{0}$ with

[3] $$\begin{array}{c} \chi_{0}=\frac{2}{\left(1-i_{0}\right)R_{0}\left(\alpha +1\right)}. \end{array}$$

(for a derivation, see *SI Appendix*, section 3.C). In Fig. 3, this is the region to the right of the red line. We find Eq. **3** fits the full numerical results remarkably well. The consequence of $\chi_{0}<1$ is that even partially altruistic individuals, who value others’ well-being less than their own, can rationally select truly Utilitarian behavior. One would not learn this without a careful formulation of partial altruism.

The main result of this paper is that the Indefinite Suppression equilibrium exists even for extremely weak altruism. Intuitively speaking, infected individuals understand that they might end up being responsible for many infections if they do not self-isolate (46). It is crucial to understand the level of partial altruism necessary to maintain this equilibrium. With this in mind, we identify two analytic expressions that together give an estimate of the location of the boundary. When the social activity reaches a value $k_{i}\approx 1/R_{0}$ the infected behavior alone is still marginally sufficient to contain the disease. We can use this to calculate the value of *χ* at which the infected adopt this marginal behavior, under the assumption that the epidemic duration is irrelevant ($t_{f}\to \infty$)

[4] $$\begin{array}{c} \chi_{\infty }=\frac{12\sqrt{i_{0}}\left(R_{0}-1\right)}{\left(3\sqrt{2}-5\sqrt{i_{0}}\right)\left(R_{0}^{2}\alpha +{\left(R_{0}-1\right)}^{2}\right)} \end{array}$$

(for the derivation, see *SI Appendix*, section 3.C). Since $\chi_{\infty }∼\sqrt{i_{0}}$, the altruism threshold can be very small. This asymptote describes the actual boundary perfectly above a certain value for $i_{0}$ for almost three orders of magnitude in $i_{0}$, see the pink line in Fig. 3.

Below this value, we find the Indefinite Suppression equilibrium persists for even smaller values of altruism than predicted by Eq. **4**. We refer to this region as the “toe.” In the toe, the finite duration of the epidemic $t_{f}$ becomes important. The intuition for understanding this is that individuals are incentivized to social distance more strongly if the end of the epidemic is in sight. By accounting for finite $t_{f}$, we obtain an expression for the altruism yielding $k_{i}=1/R_{0}$, although this employs a fairly unprincipled approximation (*SI Appendix*, section 3.C for details),

[5] $$\begin{array}{c} \chi_{t_{f}}=\frac{2i_{0}\left(R_{0}-1\right)t_{f}}{R_{0}^{2}\alpha +{\left(R_{0}-1\right)}^{2}} \end{array}$$

The expression $\chi_{t_{f}}$ is found to precisely locate the line for which $k_{i}=1/R_{0}$ but we find this is not a quantitative prediction of the boundary of the Indefinite Suppression $\chi_{c}$. However, $\chi_{t_{f}}$ still serves as an upper bound on, and rough approximant for, $\chi_{c}$. The reason for this discrepancy is that our assumptions, that $k_{i}=1/R_{0}$ and $k_{s}=1$ would hold on the boundary, were quite strict. Individuals can deviate slightly from constant behavior to stabilize the equilibrium for $\chi_{c}\leq \chi <\chi_{t_{f}}$. This is apparent in the mild social distancing of susceptibles for $\chi <\chi_{t_{f}}$ in the toe region, see Fig. 3*B*. (Since this social distancing occurs at times $t∼t_{f}$ it is not visible in Fig. 2, but see *SI Appendix*, Fig. S3) In addition, we see from Eq. **5** that $\chi_{t_{f}}∼i_{0}$. This correctly captures the scaling of the boundary $\chi_{c}$. Furthermore, the crossover of Eqs. **4** and **5** locates the top of the toe correctly.

Identification of the boundary of Indefinite Suppression is the most important outcome of this work. Our analytic expressions for this boundary represent a remarkable conceptual simplification of an extremely complex optimization problem.

#### Estimates for the critical altruism threshold in human diseases.

Estimates for the critical altruism $\chi_{c}$ can be made for a variety of diseases and locations. These estimates will be crude because our model neglects e.g. population heterogeneity and turnover, spatial effects and preexisting immunity or vaccination status, while assuming perfect information. Estimates for $R_{0}$ are available in the literature but deriving values for *α* and *ω* is more difficult. We rely here on the asymptotic estimates $\chi_{t_{f}}$ and $\chi_{\infty }$ in order to obtain upper bounds for $\chi_{c}$. These estimates are all extremely small, being in the range $10^{-5}$ to $10^{-3}$, see Table 1. Typically, the actual numerical value of $\chi_{c}$ is at least an order of magnitude smaller than those estimates would suggest. This means that very small levels of altruism should be adequate to establish an Indefinite Suppression equilibrium for all these diseases. It is hard to see how different choices of disease parameters could change this general picture.

**Table 1.** Estimates for the critical altruism threshold in human diseases

Disease	$R_{0}$	CFR	α/ω	$t_{f}$	$\chi_{\infty }$	$\chi_{t_{f}}$
COVID-19-like	3 (47)	1% (48)	4	100	$4\times 10^{-4}$	$1\times 10^{-5}$
Influenza-like	1.7 (49)	0.1% (50)	0.4	–	$4\times 10^{-3}$	–
Chickenpox-like	10 (51)	0.01% (52)	0.4	–	$2\times 10^{-5}$	–
Measles-like	15 (53)	1% (54)	40	–	$1\times 10^{-5}$	–

All reported values are rough estimates. For convenience, we assume the cost of infection *α* to be approximated by the cost of death (crudely estimated as the value of 5 statistical life years (55) for COVID-19/Influenza and 50 statistical years of life for Chickenpox/Measles) multiplied by the case fatality rate (CFR), the cost of social distancing *ω* by the total loss of median income for one week in the United Kingdom (56), and a representative $i_{0}=1\times 10^{-5}$. Estimates for $t_{f}$ are not provided for diseases where the vaccine has been available for some time.

All the results derived so far in this paper have assumed perfect information about the infection status of individuals. One might be concerned that the presence of asymptomatic infections could greatly affect these results at a qualitative level. Reassuringly:

### Indefinite Suppression Can Persist in Partially Asymptomatic Diseases.

We now consider how the self-organized behavior and disease dynamics change if a fraction of the infections are asymptomatic. To understand this, we use an SAIR compartmental model (30, 31), see *Materials and Methods* for further details. In this model, susceptible individuals who catch the disease first enter an asymptomatic/presymptomatic (but infectious) compartment. Being unaware of their infection, they adopt the same behavior as when susceptible. A proportion *σ* of the asymptomatic cases later show symptoms. They then know they are infected and behave accordingly. All individuals eventually recover. However, only those that have shown symptoms realize their immunity and cease any behavioral modification. Those who recovered while still asymptomatic keep acting as if they might still be susceptible or asymptomatic.

Within this model, the fully symptomatic case discussed in the previous section corresponds exactly to σ=1. For smaller values of *σ*, fewer individuals know that they are infected. As a result the Indefinite Suppression equilibrium becomes harder to target. This is because an increasingly smaller number of symptomatic individuals need to modify their behavior more strongly in order to suppress the disease and $\chi_{c}$ increases as a result. It is not immediately clear for what values of *σ* the Indefinite Suppression equilibrium actually exists. In the worst case scenario, individuals who believe themselves to be susceptible do not perform any social distancing (as found in the Indefinite Suppression equilibrium). In a (almost) fully susceptible population, an asymptomatic individual would then, on average, produce roughly $R_{0}\left(1-\sigma \right)$ others. Intuitively, in that scenario, complete suppression of the disease purely by symptomatically infected self-isolation becomes impossible if $R_{0}\left(1-\sigma \right)>1$. A more rigorous calculation would suggest that it is possible for the symptomatically infected individuals alone to suppress the disease by selecting $k_{i}\leq 1-\left(R_{0}-1\right)/\left(R_{0}\sigma \right)$ (*Materials and Methods*). The smallest symptomatic fraction for which this is possible is the value

**Fig. 4.**
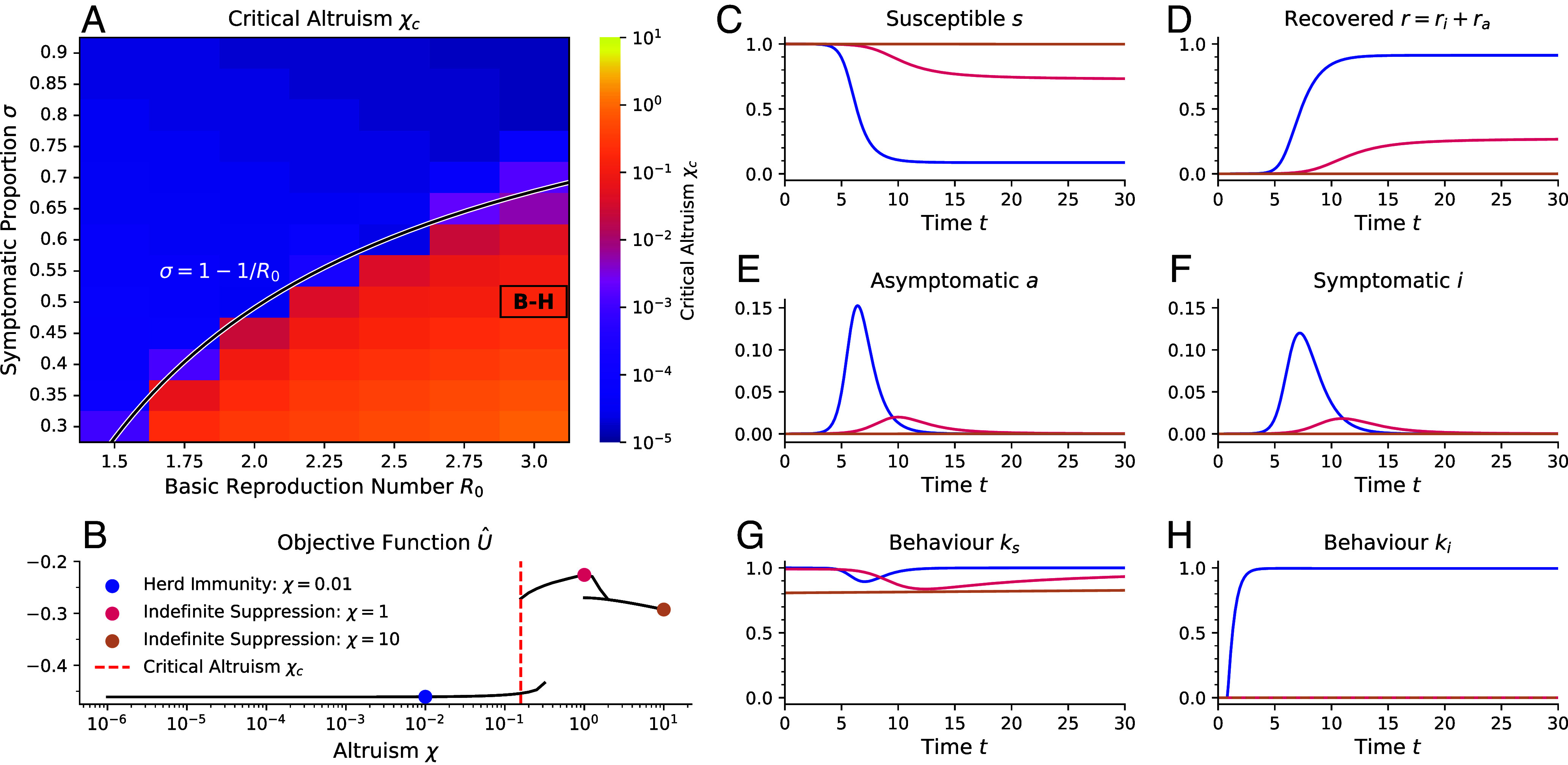
The critical altruism $\chi_{c}$ enabling Indefinite Suppression remains small as long as the fraction of symptomatic infections exceeds a threshold. (*A*) The critical value of altruism $\chi_{c}$ for which an Indefinite Suppression equilibrium exists for $\chi \geq \chi_{c}$ for asymptomatic diseases with varying values of the basic reproduction number $R_{0}$ and symptomatic proportion *σ*. For example, the value $\chi_{c}$ for the parameters indicated by the text label “B-H” is marked by the red dashed line in panel (*B*). The black line is given by $\sigma =1-1/R_{0}$, above which behaviors of $k_{s}=1,k_{i}=0$ can indefinitely suppress the disease. These results are for $\alpha =5,t_{f}=50,i_{0}=10^{-5}$. (*B*) Objective function values $\hat{U}=\;\lim_{N\to \infty }U/\left(\alpha \left(1+\chi \left(N-1\right)\right)\right)$ of all equilibria for each altruism *χ* and disease parameters of $R_{0}=3,\sigma =0.5$ [as highlighted by the text label “B-H” in panel (*A*)]. Three values of altruism are indicated by colored dots. For these, the epidemic dynamics are shown in panels (*C*–*F*), the behavior exhibited by susceptible or asymptomatic individuals $k_{s}$ is shown in panel (*G*), and the behavior of symptomatically infected individuals is shown in panel (*H*).

[6] $$\begin{array}{c} \sigma^{*}=1-1/R_{0} \end{array}$$

corresponding to $k_{i}=0$. We test these predictions by systematically calculating $\chi_{c}$ for partially asymptomatic populations numerically, see Fig. 4*A*. We find our prediction describes the data well: For $\sigma \geq \sigma^{*}$, above the black line, $\chi_{c}$ is very small, while it is much larger below the line. Indeed, above the line the behaviors and epidemic dynamics are extremely similar to the fully symptomatic limit. The main difference is that $k_{i}$ now depends on *σ*, as discussed above.

Interestingly, below the line, we find that the Indefinite Suppression equilibrium still exists but exhibits two branches, see Fig. 4*B*. In the one with a higher value of the objective function, a fraction of the population catches the disease before the infected behavior alone can suppress the disease indefinitely, see data for χ=1 in Fig. 4*B*–*H*. In the second one, the susceptible and asymptomatic individuals together indefinitely perform some social distancing. Combined with the infected behavior this then suppresses the disease, see data for χ=10 in Fig. 4*B*–*H*. It is perhaps surprising that hyperaltruistic individuals can only realize a lower utility than at χ=1. Finally, Fig. 4*B* is also an example of a situation in which there are two Nash equilibria at χ=1. Only the upper one is Utilitarian, even though in the lower branch individuals also value all population members equally. This surprising result means that an equilibrium in which one “values others as oneself” does not necessarily realize a utilitarian outcome, at least when there is a significant asymptomatic fraction.

## Discussion

Our framework enables a fundamental understanding of the effect of partial altruism on rational self-organized behavior of populations during epidemics. Here, altruism *χ* describes the degree to which others’ outcomes are valued in individual decision-making. Even though our approach is necessarily idealized, we believe it greatly improves on previous results and loose heuristics.

Reassuringly, we show that rational behavior at χ=1 can yield the social optimum. In this case, strong social distancing of infectious individuals leads to an Indefinite Suppression of the disease, provided some infections are symptomatic. Thus we also show that this optimal behavior reflects a Nash equilibrium that is stable to defection.

Surprisingly, this equilibrium behavior is not only found for perfect altruism, but can occur even when individuals feel weakly altruistic. Even then, this corresponds to near optimal outcomes.

This Indefinite Suppression equilibrium typically coexists with a Herd Immunity equilibrium characterized by a much worse outcome in which the majority of the population contracts the disease.

The quality of outcome does not vary linearly in *χ*. In fact, the outcome is almost constant on each of the equilibria with a sharp drop from Indefinite Suppression to Herd Immunity at a boundary $\chi_{c}$. The minimum value of altruism $\chi_{c}$ necessary to maintain Indefinite Suppression serves as a tipping point. For lower values of altruism, only the Herd Immunity equilibrium exists. The central result of this work is the identification of the tipping point $\chi_{c}$. Furthermore, we derived analytic estimates for this boundary that are in good agreement with the full numerical solution of our model. The threshold altruism $\chi_{c}$ has an inverse relationship with the expected infection cost and the basic reproduction number. This is intuitively reasonable: The consequences of infecting others are higher in more dangerous diseases. In addition, $\chi_{c}$ has a significant dependence on the following:

1. $i_{0}$, the infected fraction at the moment when individuals become aware of the epidemic.
2. $t_{f}$, the planning horizon, that corresponds to the expected vaccination time for humans.
3. *σ*, the fraction of infections resulting in symptoms.

As long as the symptomatic fraction is large enough, $\sigma \geq \sigma^{*}=1-1/R_{0}$, the following applies:

One can formally take the limit $t_{f}\to \infty$. In this case, we calculate the analytic estimate $\chi_{c}∼\sqrt{i_{0}}$. This reveals that the level of altruism required to stabilize Indefinite Suppression is lower the earlier the population becomes aware of a disease. We find that this estimate is in excellent agreement with the full numerical solution for an intermediate regime of $i_{0}$ that grows as $t_{f}$ increases ($10^{-3}\leq i_{0}\leq 10^{-1}$ for the parameter values shown in Figs. 1 and 3). Larger values of $i_{0}$ are likely to be of limited practical interest.

For smaller values of $i_{0}$, the finite time horizon becomes important. For values such as $i_{0}∼10^{-5}$ (corresponding to hundreds of cases in a medium sized country) and $t_{f}∼10^{2}$ disease generations (typically corresponding to 1 to 2 y) and an infection cost of α=5, the threshold is found to be in the order of $\chi_{c}∼10^{-5}$: This corresponds to an altruistic individual treating $1/\chi_{c}∼10^{5}$ members of the population as equivalent to themselves. This value of $\chi_{c}$ is a remarkably small number, revealing that an extremely small level of altruism can be sufficient to stabilize Indefinite Suppression with its vastly preferable outcomes. Quantitative estimates for $\chi_{c}$ require quantitative estimates for $R_{0}$ and the costs α,ω. However, at a qualitative level, we expect $\chi_{c}\ll 1$ to hold for most diseases of interest.

The threshold $\chi_{c}$ can be seen to be controllable by policy makers: By providing accurate and complete information about the disease to the population as early as possible, they can lower $i_{0}$ and thus $\chi_{c}$ and therefore better enable self-organization of Indefinite Suppression even when the altruism of the population is low. Also, vaccinations ought to be made available as soon as possible, not only because they have the direct effect of providing protection from infection and its consequences. They also have an indirect effect: An earlier expected vaccination time also lowers the critical altruism $\chi_{c}$ necessary for enabling the Indefinite Suppression equilibrium. For example, we estimate that $\chi_{c}$ would be reduced by about one order of magnitude by the development of a vaccine within about one year, as compared to no vaccine development, noting that $\chi_{c}\ll 1$ in either case.

In contrast, if the symptomatic fraction is too small, $\sigma <\sigma^{*}$, it becomes more challenging to maintain Indefinite Suppression. This is because self-isolation of symptomatically infectious alone is no longer sufficient to prevent a significant fraction of cases. As a result, populations with $\sigma <\sigma^{*}$ have a threshold altruism typically larger by several orders of magnitude. Either the susceptible fraction must perform indefinite social distancing or partial immunity of the population is required, here via infection. These two scenarios both correspond to equilibria that can coexist at χ=1, revealing that there are nonutilitarian equilibria accessible even at perfect altruism. Our formalism also allows us to interrogate hyperaltruistic individuals, who value others above themselves, χ>1. Perhaps surprisingly, overall outcomes can be much worse in this regime.

Given that the symptomatic fraction *σ* plays such an important role in outcomes, we note that testing for infection can increase the fraction of the population who are aware that they are infected, potentially to $\sigma >\sigma^{*}$, even if the overtly symptomatic fraction would otherwise be below this threshold. One might then expect corresponding improvements in outcomes.

Our model assumes a homogenous population. One might ask about the effect of an entirely selfish population fraction in a population of partially altruistic individuals. These individuals would not social distance while infected. In this sense they would closely resemble the asymptomatic fraction, in behavioral terms. In this interpretation, *σ* can be viewed as the fraction of the population that is aware of their infection status and altruistic. This means that our results are also robust to a moderate fraction of completely selfish individuals.

Our work reveals that the vastly preferable Indefinite Suppression equilibrium has an extremely simple behavioral profile: Infected individuals exhibit a behavioral modification that is constant in time. This response can be generated by a correspondingly simple heuristic: When infected, dramatically reduce social contacts. This represents a simple solution to a very complicated optimization problem. In the context of human disease, this heuristic would seem to be accessible to typical members of the population and can be communicated in simple term by policy makers. It could also serve as a rigorous justification for the use of such heuristics in other models (6, 57). In the case where multiple equilibria coexist the equilibrium selection problem could also be addressed by policymakers, e.g. by incorporating direct taxes or incentives to anchor the population onto the Indefinite Suppression equilibrium.

In animals, we would expect a similar behavioral heuristic to confer the same benefits. This raises the question of the extent to which such behavior is observed in nonhuman animals. Given the importance of kin selection (58) in evolutionary theory, while acknowledging that we do not investigate evolutionary stability in heterogeneous populations, such a heuristic might be expected to evolve in genetically related groups of social animals. Our altruism parameter could be expected to evolve toward the value of the genetic similarity of the group.

Indeed, there is good evidence of behavioral modification of infected individuals in perfectly altruistic eusocial animals (1). For noneusocial social animals, evidence for infectious self-isolation is sparse (1). This could indeed mean that such behavior is rare. Alternatively, it may simply reflect the fact that it is a difficult phenomenon to study. In fact, ubiquitous sickness responses, such as lethargy, may at least partially reflect the emergence of infectious self-isolation (59).

One might ask whether Indefinite Suppression might have emerged in historic human epidemics (60), acknowledging that self isolation of infectious has played an important role in the control of diseases such as HIV (61) and mpox (62). Since Indefinite Suppression would practically lead to disease elimination, one must first acknowledge an observational bias: Epidemiological data could be lacking in such cases. Historically, populations may have had a poorer understanding of human health and limited freedom to social distance, both of which could work against Indefinite Suppression. It is also worth acknowledging again that our model presents two equilibrium solutions and, even though Indefinite Suppression is vastly preferable, Herd Immunity is also a possible equilibrium outcome. Our model was designed to be simple, but could be generalized to include features such as population heterogeneity (superspreading, etc.) that could also play a role in equilibrium selection.

Our hope is that this work could help to bootstrap altruistic behavior by revealing its broad rationality (4, 5) and offer promising policy perspectives, e.g. in considering the design of interventions that leverage partial altruism.

## Materials and Methods

### Altruistic Optimal Control Methodology.

In what follows, we adopt the notation $x=\left(s,i,p_{s},p_{i}\right),k=\left(k_{s},k_{i}\right)$, $\kappa =(\kappa_{s},\kappa_{i})$. We also refer to the right hand side of Eq. **1** as *F*, so that we can write it concisely as $\dot{x}=F$.

In order to solve the constrained optimal control problem of maximizing the utility function *U*, see Eq. **2**, we recall the continuous-time Pontryagin’s Maximum/Minimum Principle (PMP) (63, 64). This describes a set of optimality conditions which an optimal trajectory $\left\{x^{⋆},\kappa^{⋆}\right\}$ must satisfy. This approach involves the construction of a Hamiltonian function H(t,x,k,κ)=u+λF and the introduction of $\lambda (t)=(\lambda_{s},\lambda_{i},\lambda_{p_{s}},\lambda_{p_{i}})$ as the costate variables (or Lagrange multipliers). The individual level costates $\lambda_{p_{s}},\lambda_{p_{i}}$ represent the shadow cost that an individual associates with being in that particular state. The population level costates $\lambda_{s},\lambda_{i}$ represent the shadow cost that an individual associates with the exogenous population being in that state. The costates allow this problem to be formulated as an unconstrained optimization problem on the Hamiltonian *H*. This method is explained further in *SI Appendix*, section 1. To summarize, these conditions amount to a set of equations for the dynamics of the state (which is given by $\dot{x}=\partial_{\lambda }H=F$), equations for the dynamics of the costate ($\dot{\lambda }=-\partial_{x}H$) and, finally, the condition that the equilibrium behavior needs to satisfy, which we call the optimality condition ($\partial_{\kappa }H=0$). The optimality condition depends on *x* and *λ*.

Since we are seeking the Nash equilibria, we set k=κ, which also then results in $s{=}_{s}$, $i{=}_{i}$.

Next, the infinite population limit N→∞ is taken, yielding a standard mean field game structure (65). In this limit, the case of χ=1 matches perfectly with the traditional Utilitarian scenario. In order to perform this limit, we found it necessary to first rescale the population level costates with the population size, such that they can be interpreted as the shadow cost that the individual associates with an average member of the population being either susceptible or infected.

The equations derived from the Hamiltonian are solved together numerically using a forward backward sweep (FBS) method (45) (*SI Appendix*, section 1.C). We explain this method in depth for the traditional self-interested and utilitarian cases in *SI Appendix*, section 2, as well as our altruistic model in *SI Appendix*, section 3.

### Altruistic Asymptomatic Disease Model.

#### Dynamics.

For the asymptomatic version of the altruistic model, we use the SAIR compartmental model (30, 31). Under this model, all susceptible individuals *s* who catch the disease go into an asymptomatic compartment *a*, and still behave according to $k_{s}$. The asymptomatic compartment includes both asymptomatic and presymptomatic individuals. It would be possible to explicitly include an additional compartment for the presymptomatic state. In order to keep the model as simple as possible, we choose to neglect this here. Infection comes from both the asymptomatic compartment and the infected (symptomatic) compartment. These types of infection are allowed to happen at different rates $\beta_{a},\beta_{i}$. Then, some proportion *σ* of the asymptomatic population start to show symptoms, know that they are infected and behave according to $k_{i}$ in the infected compartment *i*. This subpopulation then recovers and behaves according to $k_{r}$ in the $r_{i}$ compartment. On the other hand, there is a subpopulation of asymptomatics *a* who never go on to show symptoms, and thus keep behaving according to $k_{s}$. Even when they recover, entering the $r_{a}$ compartment, they still act like they are susceptible because they do not know they ever had the disease. In Fig. 4*D*, we show the total fraction of recovered, $r=r_{i}+r_{a}$. The individual version of these dynamics with states *p*_*s*_, *p*_*a*_, *p*_*i*_, $p_{r_{a}}$, $p_{r_{i}}$, and behaviors $\kappa_{s}$, $\kappa_{i}$, $\kappa_{r}$ is defined analogously. The system dynamics for both the population and individuals (with altruism), are given respectively by

[7] $$\begin{array}{cc} \frac{\mathrm{ds}}{\mathrm{dt}} & =-\beta_{a}sk_{s}\left(\left(1-\frac{1}{N}\right)ak_{s}+\frac{1}{N}p_{a}\kappa_{s}\right)\\ & -\beta_{i}sk_{s}\left(\left(1-\frac{1}{N}\right)ik_{i}+\frac{1}{N}p_{i}\kappa_{i}\right)\\ \frac{\mathrm{da}}{\mathrm{dt}} & =\beta_{a}sk_{s}\left(\left(1-\frac{1}{N}\right)ak_{s}+\frac{1}{N}p_{a}\kappa_{s}\right)\\ & +\beta_{i}sk_{s}\left(\left(1-\frac{1}{N}\right)ik_{i}+\frac{1}{N}p_{i}\kappa_{i}\right)-\left(\gamma_{\text{AR}}+\gamma_{\text{AI}}\right)a\\ \frac{\mathrm{di}}{\mathrm{dt}} & =\gamma_{\text{AI}}a-\gamma_{\text{IR}}i\\ \frac{dr_{i}}{\mathrm{dt}} & =\gamma_{\text{IR}}i\\ \frac{dr_{a}}{\mathrm{dt}} & =\gamma_{\text{AR}}a \end{array}$$

and

[8] $$\begin{array}{cc} \frac{dp_{s}}{\mathrm{dt}} & =-\beta_{a}p_{s}\kappa_{s}ak_{s}-\beta_{i}p_{s}\kappa_{s}ik_{i}\\ \frac{dp_{a}}{\mathrm{dt}} & =\beta_{a}p_{s}\kappa_{s}ak_{s}+\beta_{i}p_{s}\kappa_{s}ik_{i}-\left(\gamma_{\text{AR}}+\gamma_{\text{AI}}\right)p_{a}\\ \frac{dp_{i}}{\mathrm{dt}} & =\gamma_{\text{AI}}p_{a}-\gamma_{\text{IR}}p_{i}\\ \frac{dp_{r_{i}}}{\mathrm{dt}} & =\gamma_{\text{IR}}p_{i}\\ \frac{dp_{r_{a}}}{\mathrm{dt}} & =\gamma_{\text{AR}}p_{a} \end{array}$$

The actual force of infection terms are then dependent on the different behaviors $k_{s},k_{i},\kappa_{s},\kappa_{i}$ to affect the rate at which susceptible people move into the asymptomatic compartment. The transition rates between the other compartments are not behavior dependent, and happen at rates $\gamma_{\text{AI}}$ (Asymptomatic to Infected), $\gamma_{\text{IR}}$ (Infected to Recovered having shown symptoms) and $\gamma_{\text{AR}}$ (Asymptomatic to Recovered having not shown symptoms). Using these recovery rates, we can calculate the symptomatic proportion as $\sigma =\gamma_{\text{AI}}/\left(\gamma_{\text{AI}}+\gamma_{\text{AR}}\right)$. In order to facilitate comparison with the fully symptomatic model, we restrict ourselves to the case where $\beta_{a}=\beta_{i}=R_{0}$ and $\gamma_{\text{AR}}=\gamma_{\text{IR}}=1$, see *SI Appendix*, section 4.B for further details. This reduces the parameters of the model to $R_{0}$ and $\sigma =\gamma_{\text{AI}}/\left(\gamma_{\text{AI}}+1\right)$.

#### Utility.

These equations then also change our utility function slightly. Now, the cost of social distancing terms need to reflect all the compartments that are acting according to $k_{s},\kappa_{s}$. In addition, while the asymptomatics are infected with the disease, they are not suffering from it, so the cost of infection *α* still only applies to the infected classes *i*, *p_i_*. The utility is given by $U=\int_{0}^{t_{f}}u\;dt+U_{f}$, where

[9] $$\begin{array}{cc} u= & -\left(\alpha p_{i}+\omega_{s}\left(p_{s}+p_{a}+p_{r_{a}}\right){\left(\kappa_{s}-1\right)}^{2}+\omega_{i}p_{i}{\left(\kappa_{i}-1\right)}^{2}\right)\\ - & \chi \left(N-1\right)\left(\alpha i+\omega_{s}\left(s+a+r_{a}\right){\left(k_{s}-1\right)}^{2}+\omega_{i}i{\left(k_{i}-1\right)}^{2}\right)\\ U_{f}= & -\int_{t_{f}}^{\infty }\alpha \left(p_{i}\left(t\right)+\chi \left(\frac{1}{\epsilon }-1\right)i\left(t\right)\right)dt \end{array}$$

We solve this model with the methodology used for the fully symptomatic model, using the redefined variables $x=(s,\;a,\;i,\;r_{a},p_{s},\;p_{a},\;p_{i},\;p_{r_{a}})$, $\lambda =(\lambda_{s},\lambda_{a},\lambda_{i},\lambda_{r_{a}},\lambda_{p_{s}},\lambda_{p_{a}},\lambda_{p_{i}},\lambda_{p_{r_{a}}})$, where we drop the compartments $r_{i},\;p_{r_{i}}$ for brevity, as they play no role in the utility or disease dynamics.

#### Threshold on the asymptomatic fraction.

Within our asymptomatic model the disease is being partially propagated by an asymptomatic fraction behaving as if susceptible. To what extent can the infected population then control the disease purely through their own behavior? In order to achieve a full Indefinite Suppression, $\dot{a}<0$ is required. So at a Nash equilibrium, this equates to

[10] $$\begin{array}{c} R_{0}sak_{s}^{2}+R_{0}sik_{s}k_{i}-\left(1+\gamma_{\text{AI}}\right)a<0. \end{array}$$

If we assume the symptomatically infected to select constant behavior while others adopt behavior $k_{s}=1$ then, with i=aσ/(1−σ) and s≈1, this condition reduces to

[11] $$\begin{array}{c} R_{0}a+R_{0}k_{i}a\frac{\sigma }{1-\sigma }-\left(\frac{1}{1-\sigma }\right)a<0. \end{array}$$

providing a prediction for $k_{i}$,

[12] $$\begin{array}{c} k_{i}\leq 1-\left(R_{0}-1\right)/\left(R_{0}\sigma \right) \end{array}$$

In the special case of the most extreme distancing possible $k_{i}=0$ we obtain the condition

[13] $$\begin{array}{c} \sigma >\sigma^{*}=\frac{R_{0}-1}{R_{0}} \end{array}$$

for when it is possible for purely infected individuals to indefinitely suppress the disease.

## Supplementary Material

Appendix 01 (PDF)

## Data Availability

There are no data underlying this work.
