# Supplementary material for: The theory of epidemics with altruism
Source: Proc Natl Acad Sci U S A. 2026 Feb 27;123(9):e2518893123. doi: 10.1073/pnas.2518893123 (PMC12956880; doi:10.1073/pnas.2518893123)
Supplement: Supplementary file 1 — Appendix 01 (PDF) [file pnas.2518893123.sapp.pdf]

PNAS

Supporting Information for

The Theory of Epidemics with Altruism

Mark P. Lynch, Simon K. Schnyder, John J. Molina, Ryoichi Yamamoto, Matthew S. Turner

Simon K. Schnyder
E-mail: skschnyder@gmail.com
Matthew S. Turner
E-mail: m.s.turner@warwick.ac.uk

This PDF file includes:

Supporting text
Figs. S1 to S6
SI References

Contents

1 Theoretical Background **S2**
A Calculus of Variations S2
A.1 Euler-Lagrange Equations S2
A.2 Constraints S3
B Optimal control theory S4
B.1 Bounded Controls S5
C Forward Backwards Sweep S5

2 Selfish and Utilitarian Limits **S6**
A Non-altruistic Epidemic Dynamics S6
B General Utility S7
C Selfish Individuals S7
C.1 Theory S7
C.2 Cleanup Term under perfect vaccination S8
D Utilitarian Maximum S9
D.1 Theory S9

3 Altruistic Model **S10**
A Motivation S10
B Model Description S10
B.1 Dynamics S10
B.2 Theory S11
B.3 Selfish Limit S13
B.4 Utilitarian Limit S14
C Analytic estimates for behaviour S14
C.1 Analytic solution for constant behaviour S14
C.2 Nash equilibrium S15
D Creating the Heatmaps S17

4 Altruistic Asymptomatic Model **S17**
A Model Description S18
A.1 Asymptomatic Cleanup Term under perfect vaccination S18
A.2 Theory S18
B Calculating the Basic Reproduction Number S19
C Obtaining χ_c S21

5 Supporting Results **S21**
A Self Interested Results S21
B Utilitarian Results S22
C Coexistence of Both Equilibria S22
D Varying Parameters S22

1. Theoretical Background

We first provide more details regarding how we can set up and solve optimal control problems of the form used in the main paper. Whilst the main crux of our model relies on the interplay between the individuals and the population, we will present a generalised framework for these sorts of problems. We then apply this to an epidemic setting and derive the traditional self-interested and Utilitarian cases in section 2.C and section 2.D so that we can show how our altruistic model interpolates between these two in section 3.

A. Calculus of Variations.

A.1. Euler-Lagrange Equations. Let $q(t)$ be a path function for a given dynamical system, which specifies the coordinates q as a function of time. We characterize the paths in terms of an *Action* S, a functional $S[q]$, which is defined as the time integral of a *Lagrangian* L, itself a function of time t, coordinates q, and velocities $\dot{q} = \mathrm{d}q/\mathrm{d}t$, plus an (optional) so-called cleanup term, which here depends only on the value of the path at the end point at t_f,

$$S[q](t_1, t_2) = \int_{t_1}^{t_2} L(t, q(t), \dot{q}(t))\,\mathrm{d}t + \phi(t, q(t_2)) \quad \text{[S1]}$$

$$= \int_{t_1}^{t_2} L\,\mathrm{d}t + \phi(t_2, q(t_2)) \quad \text{[S2]}$$

Let h be a variation of the path, such that $q \to q + h$, the corresponding variation in S, to first-order, is (1)

$$\delta_h S[q](t_1, t_2) = \big(S[q+h] - S[q]\big)(t_1, t_2) \tag{S3}$$
$$= \int_{t_1}^{t_2} \left[L(t, q+h, \dot{q}+\dot{h}) - L(t, q, \dot{q})\right] + \left[\phi(t, q+h) - \phi(t, q)\right]\Big|_{t=t_2} \tag{S4}$$
$$= \int_{t_1}^{t_2} \left[(\partial_q L)\, h + (\partial_{\dot{q}} L)\, \dot{h}\right] \mathrm{d}t + (\partial_q \phi)\, h\big|_{t_2} + \mathcal{O}(h^2) \tag{S5}$$

Using integration by parts on the second term in the integrand, and dropping terms of order higher than $\mathcal{O}(h^2)$, we have

$$\delta_h S[q](t_1, t_2) = \int_{t_1}^{t_2} \left(\partial_q L - \frac{\mathrm{d}}{\mathrm{d}t}\partial_{\dot{q}} L\right) h\, \mathrm{d}t + (\partial_{\dot{q}} L + \partial_q \phi)\, h\big|_{t_2} - (\partial_{\dot{q}} L)\, h\Big|_{t_1} \tag{S6}$$

The optimal path q is that which extremizes the Action, such that $\delta_h S[q] = 0$. Since the variations h are arbitrary, except possibly at the end-points, each of the terms in Eq. S6 (as well as the integrand) must vanish exactly. We thus obtain the following set of equations (1)

$$\frac{\mathrm{d}}{\mathrm{d}t}\partial_{\dot{q}} L - \partial_q L = 0 \tag{S7}$$
$$(\partial_{\dot{q}} L + \partial_q \phi)\, h\big|_{t_2} = 0 \tag{S8}$$
$$(\partial_{\dot{q}} L)\, h\big|_{t_1} = 0 \tag{S9}$$

Eqs. S7 are the Euler-Lagrange (EL) equations, Eqs. S8-S9 provide additional boundary conditions, which may or may not be required depending on whether or not the end-points are fixed.

A.2. Constraints. Consider the following constrained optimization problem

$$\delta_h S[q](t_1, t_2) = 0 \tag{S10}$$
$$\text{such that} \quad \psi(t, q, \dot{q}) = 0. \tag{S11}$$

If ψ is an integral or holonomic constraint (2), this constrained optimization can be expressed as an unconstrained optimization, by considering an augmented system. Let $q' = (q, \lambda)$ be the coordinates for this new system, where λ are additional degrees of freedom (one for each constrained coordinate). The solution to the (constrained) optimization for q, is equivalent to the solution of the (unconstrained) optimization for q' given by(2)

$$\delta_{h'} S'[q](t_1, t_2) = \delta_{h'} \left[\int_{t_1}^{t_2} L'\, \mathrm{d}t + \phi(t_2, q(t_2))\right] = 0 \tag{S12}$$
$$L'(t, q', \dot{q}') = L(t, q, \dot{q}) + \lambda \cdot \psi(t, q, \dot{q}) \tag{S13}$$

The EL equations for the original q coordinates are

$$\frac{\mathrm{d}}{\mathrm{d}t}\partial_{\dot{q}} L' - \partial_q L' = 0 \tag{S14}$$

whereas the EL equations for the λ coordinates simply reproduce the constraint equations

$$\frac{\mathrm{d}}{\mathrm{d}t}\partial_{\dot{\lambda}} L' - \partial_\lambda L' = -\psi = 0 \tag{S15}$$

Since the Lagrangian only depends on λ and not $\dot{\lambda}$, and the cleanup term is independent of λ, only the boundary conditions for the q degrees of freedom remain

$$\left(\partial_{\dot{q}} L' + \partial_q \phi\right) h\Big|_{t_2} = 0 \tag{S16}$$

We have ignored the additional boundary condition at t_0, since in what follows we consider problems with fixed initial state, i.e., $q(t_0) = q_0$, such that $h(t_0) = 0$.

B. Optimal control theory. Optimal control theory can be considered as a special case of the extremization problem detailed in the previous section. In particular, consider a dynamical system with coordinates $x(t)$ that satisfy the following ordinary differential equations

$$\dot{x}(t) = F(t, x(t), c(t), \xi(t)) = F_\xi(t, x(t), c(t)), \qquad x(0) = x_0 \tag{S17}$$

$$\dot{x}^i(t) = F^i(t, x(t), c(t), \xi(t)) = F^i_\xi(t, x(t), c(t)) \tag{S18}$$

where c are the co-called control variables, which can be independently specified, and ξ is an arbitrary external or exogenous field. While the system dynamics are constrained by $\dot{x} = F$, the time-evolution of the system can be controlled by specifying c. The question then, is to find the optimal control, i.e., that which extremizes the cost functional U

$$U_\xi[x, c](t_0, t_f) = \int_{t_0}^{t_f} u_\xi(t, x(t), c(t))\,\mathrm{d}t + U_f(t_f, x(t_f)) \tag{S19}$$

Here, the degrees of freedom are (x, c), the payoff function u plays the role of the Lagrangian, U_f is the cleanup term.

As detailed above, the solution to this constrained optimization problem can be found by introducing additional degrees of freedom, the co-state variables λ, and defining an augmented Lagrangian

$$q = (x, \lambda, c) = \left(\left(x^1, \ldots, x^n\right), (\lambda_1, \ldots, \lambda_n), \left(c^1, \ldots, c^m\right)\right) \tag{S20}$$

$$L_\xi(t, q, \dot{q}) = u_\xi(t, x, c) + \lambda \cdot (F_\xi(t, x, c) - \dot{x}) \tag{S21}$$

$$= u_\xi(t, x, c) + \sum_{i=1}^{n} \lambda_i \left(F^i_\xi(t, x, c) - \dot{x}^i\right) \tag{S22}$$

where we assume that x (λ) are n-dimensional, while c is m-dimensional. Define H to be the part of the Lagrangian with no velocity dependence

$$L_\xi(t, q, \dot{q}) = L_\xi(t; x, \lambda, c; \dot{x}, \cdot, \cdot) \tag{S23}$$

$$= [u_\xi(t, x, c) + \lambda \cdot F_\xi(t, x, c)] - \lambda \cdot \dot{x} \tag{S24}$$

$$= H_\xi(t, x, \lambda, c) - \lambda \cdot \dot{x} \tag{S25}$$

such that

$$H_\xi(t, q) = H_\xi(t, x, \lambda, c) = u_\xi(t, x, c) + \lambda \cdot F_\xi(t, x, c) \tag{S26}$$

$$\partial_x H_\xi = \partial_x L_\xi \tag{S27}$$

$$\partial_c H_\xi = \partial_c L_\xi \tag{S28}$$

$$\partial_{\dot{q}} H_\xi = 0 \tag{S29}$$

In contrast to the general setup considered above, here the original Lagrangian u is independent of velocity, such that the augmented Lagrangian only depends on $\dot{x}$ due to the introduction of the constraint term. Thus, the H turns out to be the Hamiltonian corresponding to the Lagrangian L (i.e., the Legendre transform of L with respect to $\dot{x}$)(2), with λ interpreted as the momenta conjugate to x. Because of this, the Lagrangian and Hamiltonian equations are not only equivalent, but identical.

The resulting EL equations, and boundary conditions, are

$$\frac{\mathrm{d}}{\mathrm{d}t}\frac{\partial L_\xi}{\partial \dot{q}^\alpha} = \frac{\partial L_\xi}{\partial q^\alpha} \qquad \alpha = 1, \ldots, 2n + m \tag{S30}$$

$$\left.\left(\partial_{\dot{x}^i} L_\xi + \partial_{x^i} U_f\right)\right|_{t_f} = 0 \qquad i = 1, \ldots, n \tag{S31}$$

The EL equations for the x degrees of freedom ($\alpha = 1, \cdots, n$) provide the dynamical equations for the costate λ variables, since $\partial_{\dot{x}^i} L = -\lambda_i$

$$\dot{\lambda}_i(t) = -\partial_{x^i} L_\xi(t, x, \lambda, c; \dot{x}, \cdot, \cdot) = -\partial_{x^i} H_\xi(t, x, \lambda, c) \tag{S32}$$

$$\lambda_i(t_f) = \partial_{x^i} U_f(t_f, x(t_f)) \tag{S33}$$

As usual, the EL equations for the costate λ degrees of freedom simply restate the dynamical constraint

$$\dot{x}^i(t) = F^i_\xi(t, x(t), c(t)) \tag{S34}$$

Finally, the EL equations for the control c degrees of freedom provide an implicit equation for the optimal control at each instant in time

$$\left.\frac{\partial L_\xi}{\partial c^j}\right|_{c=c^\star} = \left.\frac{\partial H_\xi}{\partial c^j}\right|_{c=c^\star} = 0 \tag{S35}$$

To summarize, the solution to an optimal control problem of the form

$$c^{\star}(t) = \arg\max_{c} \left[\int_{t_0}^{t_f} u(t, x(t), c(t)) + U_f(t_f, x(t_f)) \right] \quad \text{[S36]}$$

$$\text{such that} \quad \dot{x}(t) = F(t, x(t), c(t)) \quad \text{[S37]}$$

$$x(t_0) = x_0 \quad \text{[S38]}$$

can be found by integrating the following set of ordinary differential equations for the state x and costate λ

$$\dot{x}(t) = F(t, x(t), c^{\star}(t)) \quad \text{[S39]}$$

$$\dot{\lambda}(t) = -\partial_{x^i} H(t, x(t), \lambda(t), c^{\star}(t)) \quad \text{[S40]}$$

$$H(t, x, \lambda, c) = u(t, x, c) + \lambda \cdot F(t, x, c) \quad \text{[S41]}$$

with mixed boundary conditions for the initial state $x(t_0)$ and the final co-state $\lambda(t_f)$

$$x(t_0) = x_0 \quad \text{[S42]}$$

$$\lambda(t_f) = \partial_{x^i} U_f(t_f, x(t_f)) \quad \text{[S43]}$$

under the optimality condition for the control c

$$\left(\partial_c H\right)(t, x, \lambda, c^{\star}) = 0 \quad \text{[S44]}$$

which we assume allows us to solve for the optimal control $c^{\star}$ as a function of time t, state x, and costate λ

$$c^{\star}(t) = c^{\star}(t, x(t), \lambda(t)) \quad \text{[S45]}$$

To accelerate convergence of our numerical solvers, it is convenient to express the solution to this optimal control problem as a fixed-point problem, i.e., for given time t, state x, and costate λ, we define

$$c^{\star} = \mathcal{C}(c^{\star}) \equiv \mathcal{C}(c^{\star}; t, x, \lambda) \quad \text{[S46]}$$

In Eqs. S36-S45 we have dropped the dependence on the external field ξ, as it plays no role in deriving the equations or the boundary conditions. However, it will be useful in what follows, when we consider the solutions to mean-field models and are required to enforce consistency between individual and collective degrees of freedom.

B.1. Bounded Controls. It may be necessary in some systems to bound the control, i.e., to constrain c within a set of allowed values $c \in \mathcal{A}$. In these situations, Pontryagin's Principle provides the generalization to the EL equations derived above (3). In particular, the optimality condition $(\partial_{c^j} H)(t, x, \lambda, c^{\star}) = 0$ is replaced with

$$c^{\star} = \arg\max_{c \in \mathcal{A}} H(t, x, \lambda, c) \quad \text{[S47]}$$

Assuming the control is bounded as $C_0^j \leq c^j \leq C_1^j$, the constrained solution $c^{\star}$ satisfies

$$\begin{cases} (c^{\star})^j = C_0^j & \text{if} \;\; \partial_{c^j} H|_{c=c^{\star}} < 0 \\ C_0^j < (c^{\star})^j < C_1^j & \text{if} \;\; \partial_{c^j} H|_{c=c^{\star}} = 0 \\ (c^{\star})^j = C_1^j & \text{if} \;\; \partial_{c^j} H|_{c=c^{\star}} > 0. \end{cases} \quad \text{[S48]}$$

In practice, we can simply truncate the optimal control solution to be at the bounds (4)

$$\hat{c} = \arg\max_{c} H(t, x, \lambda, c) \quad \text{[S49]}$$

$$(c^{\star})^j = \begin{cases} C_0^j & \text{if } \hat{c}^j < C_0^j \\ \hat{c}^j & \text{if } C_0 \leq \hat{c}^j \leq C_1^j \\ C_1^j & \text{if } \hat{c}^j > C_1^j \end{cases} \quad \text{[S50]}$$

C. Forward Backwards Sweep. To solve the optimality equations, we use a version of the Forward-Backward Sweep Method (FBS Method) presented in (4). We assume an initial condition for the state x but a final condition for the costate λ (given in Eq. S33). Also note that $\dot{x}$ does not depend explicitly on the costate λ, only on time t, the state x and the control c (see Eq. S39). The FBS Method uses this information very intuitively.

0. Make some initial guess for the control $c'(t)$ over the time interval $[t_0, t_f]$.

1. Using the initial condition x_0 (see Eq. S42) and the current control $c'(t)$, solve $\dot{x} = F(t, x, c')$ (see Eq. S39) forwards in time to obtain an estimate for the state trajectory $x'(t)$.

2. Using the final time condition $\lambda(t_f)$ (see Eq. S43), and the current values of the state and control, $x'(t)$ and $c'(t)$, solve $\dot{\lambda} = -\partial_x H(t, x', c', \lambda)$ (see Eq. S40) backwards in time, to obtain an estimate for the costate trajectory λ'.

3. Use the current estimates for the state x' and costate λ' within a suitable *FBS Update Rule* (applying the necessary bounds), to update the guess for the control to c''. Here we use the fixed-point representation of Eq. S46, $c'' = \mathcal{C}(c'; t, x', \lambda')$.

4. Use a convex combination of the old control $c^{\text{old}} = c'$ and the new estimate c'', to determine the value to use for the next iteration, i.e., $c^{\text{new}} = (1-\nu)c^{\text{old}} + \nu c''$, with $0 < \nu < 1$.

5. Check for Convergence. If the control values $c^{\text{new}}, c^{\text{old}}$ are negligibly close, then output the current state, control and costate. Otherwise, set $c' = c^{\text{new}}$ and repeat from Step 1.

In Step 4, the degree to which we mix in the new guess for the control is specified by the *mixing weight* ν. The convergence criteria used in Step 5 checks that the controls of two sequential iterations $c^{(n)}$ and $c^{(n+1)}$ satisfy the following inequality for all times t

$$|c_j^{(n+1)}(t) - c_j^{(n)}(t)| < \delta \quad \forall j, t \tag{S51}$$

where $\delta \ll 1$ and $\nu < 1$. When this absolute convergence criteria is satisfied by the control at every point in time, then convergence is said to have been reached. Note that the state and control at iteration n are mixed into the values at iteration $n+1$ according to step 4 above. This means the value of δ corresponds to the fractional change of the new (unmixed) estimate of the control variables relative to the previous one. We can therefore rewrite the above condition (on the controls) as

$$|c_j^{(n)}(t) - c_{\text{new},j}^{(n)}(t)| < \frac{\delta}{\nu} \quad \forall j, t \tag{S52}$$

As default, we use $\nu = 1 \times 10^{-3}, \delta = 1 \times 10^{-6}$, implying that convergence stops when the maximal change of the control is less than 0.1%. One can keep the same percentage and decrease both ν and δ by the same order of magnitude if finer precision in the control value is needed.

In some cases, where stricter convergence was required, we used a relative convergence criterion given by

$$\frac{|c_j^{(n+1)}(t) - c_j^{(n)}(t)|}{|c_j^{(n+1)}(t)|} < \delta \quad \forall j, t. \tag{S53}$$

Convergence is difficult to achieve if the control values are close to zero, but we found that it provides more accurate results for control values greater than $\approx 1 \times 10^{-2}$. Here, we cannot separate out the mixing rate ν as in Eq. S52, but as ν decreases, we can make a similar approximation of the control varying by less than 0.1%. Since control values are typically less than 1, we counteract the effect of denominator in the criterion by setting δ an order of magnitude less.

2. Selfish and Utilitarian Limits

A. Non-altruistic Epidemic Dynamics. We consider an epidemic that follows a standard SIR compartmentalised model (5), which tracks the proportion of the population at any given time that is in either the susceptible $s(t)$, infected $i(t)$ or recovered $r(t)$ classes. The dynamics obey the system of equations

$$\begin{aligned} \dot{s} &= -\beta(.)si \\ \dot{i} &= \beta(.)si - \gamma i \\ \dot{r} &= \gamma i \end{aligned} \tag{S54}$$

with initial values $s(0) = 1 - i_0$ and $i(0) = i_0$. Whenever variables are defined as time-dependent, we drop these functional dependencies on time for brevity. The rate of recovery γ is assumed to be constant but the transmission rate $\beta(.)$ is variable dependent on both the behaviour of the population and the infectiousness of the disease. In a similar way to Bethune et. al (6), the behaviour of the population is said to depend on their infection status. The susceptible population is allowed to behave differently to the infected population.

Let $k_s(t), k_i(t)$ be the respective behaviour of the susceptible and individual populations. We interpret these behaviours as the amount of social distancing that the respective sub-populations perform. Behaviours of $k_s, k_i = 1$ are taken as normal population behaviour under no social distancing, and a reduction in k_s, k_i implies social distancing performed by that particular sub-population. By taking time to be in units of recovery rate γ, the behavioural population level dynamics are given by

$$\begin{aligned} \dot{s} &= -R_0 k_s k_i si \\ \dot{i} &= R_0 k_s k_i si - i \\ \dot{r} &= i. \end{aligned} \tag{S55}$$

Here we introduce R_0, the Basic Reproduction Number. This is defined as the average number of secondary infections caused by the average primary infection in a totally susceptible population. This will be the case under no social distancing, but either

sub-population can affect the rate of infection through social distancing. So, like Bethune et. al (6), we take the product of R_0 and the behaviours to be the transmission rate ($\beta(x, y) = R_0 xy$). This gives us the *effective R number.*

We make special note that the recovered individuals have their own behaviour too, denoted $k_r(t)$. However, the behaviour of recovered individuals has no effect on either the rate of infection or the rate of recovery. Since we later assume there is no inherent cost to being recovered, but there is a cost to social distancing, we omit k_r from any of our formulations by simply assuming $k_r(t) = 1$.

On an individual level, no single person can directly control the behaviour of the population. An individual can, however, change their own behaviour. In the absence of government policy, any change is likely to be influenced by the behaviour of the population as well as the current state of the epidemic. An individual can either be susceptible, infected or recovered from the disease, and they move between these classes thanks to stochastic events of infection and recovery. Therefore we calculate the expected probability $p_j(t)$ that an individual is in compartment j. They can influence their transition rates between these classes by controlling $\kappa_j(t)$, their behaviour whilst in compartment j. These individual dynamics are given by

$$\begin{aligned}\dot{p_s} &= -R_0\kappa_s k_i p_s i\\ \dot{p_i} &= R_0\kappa_s k_i p_s i - p_i\\ \dot{p_r} &= p_i,\end{aligned} \qquad \text{[S56]}$$

Susceptible individuals become infected through interactions with the infected population. The combined system dynamics in Eqs. S55-S56 assume that an individual cannot infect themselves, nor can they infect the population. The population is taken to be so large that the impact of any one infectious individual is negligible. The SIR dynamics assume no reinfection, and so the recovered individuals has no impact on the course of the disease, thus we omit state (as well as the behaviour) of the recovered classes from here on. Within this approximation, the full dynamics for the state of the combined system (individual and population) is given by

$$\begin{aligned}\dot{s} &= -R_0 k_s k_i s i\\ \dot{i} &= R_0 k_s k_i s i - i\\ \dot{p_s} &= -R_0\kappa_s k_i p_s i\\ \dot{p_i} &= R_0\kappa_s k_i p_s i - p_i,\end{aligned} \qquad \text{[S57]}$$

In the sections that follow, we use as vectorised shorthand $\theta = (s, i), k = (k_s, k_i), p = (p_s, p_i), \kappa = (\kappa_s, \kappa_i)$.

B. General Utility. The simple utility function we use takes an integral of some payoff function over the whole epidemic. This represents individuals seeking to take their future costs into account when making decisions in the present. The form of such a utility is given by the following generic form

$$\begin{aligned}U &= \int_0^{t_f} u\ dt + U_f\\ u &= p_s(t)u_s(t) + p_i(t)u_i(t).\end{aligned} \qquad \text{[S58]}$$

The integrand u shows that there are certain costs associated with being in each compartment $u_j(t)$. The individual only suffers these costs if they are in that respective compartment, hence the products with $p_j(t)$.

Many economic models of this form will include some discounting term in the payoff (7, 8), where individuals can see the impact that their decisions will have a few days in the future, but not the whole trajectory of the epidemic. For brevity, we exclude such a term from our formulation, although one could investigate the affect of discounting with the altruistic model presented in this paper. We leave that for future work.

With an SIR type disease, assuming no re-infection or multiple strains, the system state reaches a point where little to no further infections are occurring, and so levels of susceptible and recovered individuals remain virtually constant at late times. One could take the above integral over an infinite time horizon. We, however, truncate our integral at some large final time t_f where the disease in no longer sufficiently spreading. A cleanup term U_f can then be defined for costs after this final time t_f. There are multiple assumptions we could make about this end-time. We say that a perfect vaccine is found and is rolled out on mass to everyone in the population. This reduces the proportion of susceptibles immediately to zero and the disease no longer spreads. The proportion of infected who are suffering the costs of being infected then decays exponentially over time.

C. Selfish Individuals.

C.1. Theory. We consider the problem of optimizing for the behavior of purely selfish individuals. Within the general formalism presented in section 1.B, we identify the dynamical system and the control with the individual state and behavior, $x = p$ and $c = \kappa$, respectively, with the population state and control the external fields $\xi = (\theta, k)$. The dynamical constraints on $\dot{x}$ introduce two costate variables λ. Thus, we have

$$x = p = (p_s, p_i) \qquad \text{[S59]}$$

$$c = \kappa = (\kappa_s, \kappa_i) \qquad \text{[S60]}$$

$$\lambda = (\lambda_s, \lambda_i) \qquad \text{[S61]}$$

$$\xi = (\theta, k) = ((s, i), (k_s, k_i)) \qquad \text{[S62]}$$

where the state dynamics is constrained to be the individual SIR dynamics

$$F_\xi(t,x,c) = F_\xi(t,p,\kappa) = \begin{pmatrix} F_\xi^{p_s} \\ F_\xi^{p_i} \end{pmatrix} = \begin{pmatrix} -R_0\kappa_s k_i p_s i \\ R_0\kappa_s k_i p_s i - p_i \end{pmatrix} \quad \text{[S63]}$$

We assume a basic payoff function of the form

$$u_\xi(t,x,c) = u_{\theta,k}(t,p,\kappa) = -\alpha p_i - \omega_s p_s(\kappa_s - 1)^2 - \omega_i p_i(\kappa_i - 1)^2 \quad \text{[S64]}$$

which has two basic costs associated with an epidemic. Firstly, there is a cost to being infected by a disease. Such a cost is given by α and is only associated with the infectious compartment. More generally, this cost of infection could vary with different factors, such as the current proportion of infected individuals, but for simplicity we keep this a constant value for the disease. Secondly, there is a cost to social distancing beyond the normal population behavior. The behaviour of the population in the absence of any disease is scaled to be 1. Individuals who are either susceptible or infected are said to incur some squared cost from deviating from this social norm, scaled by the respective cost of social distancing in that compartment (here ω_s or ω_i). One could consider different costs to social distancing, such as a logarithmic cost which gives an infinite cost for setting behaviour to zero. We use a squared cost similar to Makris and Toxvaerd (9).

The Hamiltonian is therefore given by

$$\begin{aligned} H_\xi(t,p,\lambda,\kappa) &= u_\xi(t,p,\kappa) + \lambda\cdot F_\xi(t,p,\kappa) = u - \Delta R_0\kappa_s k_i p_s i - \lambda_i p_i \\ \Delta &= \lambda_s - \lambda_i \end{aligned} \quad \text{[S65]}$$

The optimimal control $\kappa^\star$ is obtained from the optimality condition ($\partial_\kappa H_\xi = 0$) as

$$\begin{pmatrix} 2\omega_s p_s & 0 \\ 0 & 2\omega_i p_i \end{pmatrix} \cdot \begin{pmatrix} \kappa_s^\star \\ \kappa_i^\star \end{pmatrix} = 2\begin{pmatrix} \omega_s p_s - \frac{R_0}{2} i k_i p_s \Delta \\ \omega_i p_i \end{pmatrix} \quad \text{[S66]}$$

$$\begin{pmatrix} \kappa_s^\star \\ \kappa_i^\star \end{pmatrix} = \begin{pmatrix} 1 - \frac{R_0}{2\omega_s} i k_i \Delta \\ 1 \end{pmatrix} \quad \text{[S67]}$$

From this, we see that the behaviour of infected individuals never deviates from the baseline population behaviour $\kappa_i = 1$. The interpretation here being that if an individual is acting selfishly, and they are already suffering the cost of being infected, there is no reason to suffer the further cost of social distancing to protect yourself or anyone else from any further costs.

Within a mean-field model of identical individuals, we target the Nash equilibrium solution by requiring self-consistency between the population and the individual, i.e., $\theta = p$ and $k = \kappa$. Thus, the Nash solution for the optimal behavior is

$$k^\star = k^\star(t,\theta,\lambda) = \kappa_\xi^\star(t,p,\lambda)\big|_{\theta=p,k=\kappa} \quad \text{[S68]}$$

$$= \begin{pmatrix} 1 - \frac{R_0}{2\omega_s} i k_i^\star \Delta \\ 1 \end{pmatrix} = \begin{pmatrix} 1 - \frac{R_0}{2\omega_s} i\Delta \\ 1 \end{pmatrix} \quad \text{[S69]}$$

with state and costate dynamics given by

$$\dot\lambda = -(\partial_p H_\xi)(t,p,\lambda,\kappa^\star)|_{\theta=p,k=\kappa} \quad \text{[S70]}$$

$$= \begin{pmatrix} \omega_s(k_s^\star - 1)^2 + \Delta R_0 k_s^\star k_i^\star i \\ \alpha + \omega_i(k_i^\star - 1)^2 + \lambda_i \end{pmatrix} \quad \text{[S71]}$$

$$\dot p = F_\xi(t,p,\kappa_\xi^\star)\big|_{\theta=p,k=\kappa} \quad \text{[S72]}$$

$$= \begin{pmatrix} -R_0 k_s^\star k_i^\star s i \\ -R_0 k_s^\star k_i^\star s i - i \end{pmatrix} \quad \text{[S73]}$$

and end-time boundary conditions (the appropriate cleanup cost U_f from which these are derived is discussed in section C.2)

$$\lambda(t_f) = \partial_p U_f(t_f, p(t_f)) = \begin{pmatrix} 0 \\ -\alpha \end{pmatrix} \quad \text{[S74]}$$

C.2. Cleanup Term under perfect vaccination. At the end-time t_f, we make the assumption that a 'perfect vaccine' becomes available which instantly immunises all susceptible individuals, such that $p_s(t > t_f) = s(t > t_f) = 0$. This immediately reduces the disease dynamics to the equations

$$\begin{aligned} \dot i &= -i \\ \dot p_i &= -p_i \end{aligned} \quad \text{[S75]}$$

The solution to this system of equations gives the fraction of infected, and the probability of an individual to be infected, after time t_f, as

$$\begin{aligned} i(t > t_f) &= i_f \exp[-(t - t_f)] \\ p_i(t > t_f) &= p_{i,f} \exp[-(t - t_f)]. \end{aligned} \quad \text{[S76]}$$

where $i_f = i(t_f), p_{i,f} = p_i(t_f)$. In this setting, since there is no risk of becoming infected, and no risk of passing the infection onto others, there is no reason to socially distance. Thus, $k_j(t > t_f) = \kappa_j(t > t_f) = 1$ $(j = s, i)$. For the payoff in Eq. S58, the late-time utility U_f is given by

$$U_f = \int_{t_f}^{\infty} u_i(p_s = 0, p_i, \kappa = (1,1)) p_i dt \qquad \text{[S77]}$$

where u_i is a function only of p, since $\kappa = (1,1)$. By substituting Eq. S75 and using the chain rule we get a general expression for the cleanup term

$$\begin{aligned} U_f(t_f, p(t_f)) &= \int_{t_f}^{\infty} u_i(p_i) p_i dt \\ &= -\int_{t_f}^{\infty} u_i(p_i)\dot{p}_i dt \\ &= \int_0^{p_{i,f}} u_i(p_i) dp_i. \end{aligned} \qquad \text{[S78]}$$

At this point, we use the explicit form of the payoff $u_i = -\alpha_p$ to derive the cleanup term as $U_f = -\alpha_p p_{i,f}$. Using Eq. S43, we derive the end-time boundary conditions

$$\lambda(t_f) = \partial_p U_f(t_f, p(t_f)) = \begin{pmatrix} 0 \\ -\alpha \end{pmatrix}. \qquad \text{[S79]}$$

Optimal behaviours that solve this optimal control problem are shown in Supplementary Text 5.A.

D. Utilitarian Maximum.

D.1. Theory. The Nash Equilibrium does not necessarily give the best possible utility value for the entire population, which we refer to as the Utilitarian Maximum. In order to find this solution, we consider the SIR dynamics for the population, as given in Eq. S55, and assume that the population behaviour can be controlled directly. In other words, we now take as state and control variables the population state and behavior, $x = \theta$ and $c = k$, respectively,

$$x = \theta = (s, i) \qquad \text{[S80]}$$
$$c = k = (k_s, k_i) \qquad \text{[S81]}$$
$$\lambda = (\lambda_s, \lambda_i) \qquad \text{[S82]}$$
$$\xi = 0 \qquad \text{[S83]}$$

where the costate variables are here used to contrain the population dynamics to be SIR

$$F(t, x, c) = F(t, \theta, k) = \begin{pmatrix} F^s(t, \theta, k) \\ F^i(t, \theta, k) \end{pmatrix} = \begin{pmatrix} -R_0 k_s k_i s i \\ R_0 k_s k_i s i - i \end{pmatrix} \qquad \text{[S84]}$$

Note that in this case, there is no need for any exogenous field ξ. We consider a similar payoff function as for the selfish individuals (Eq. S64), except now costs are taken with respect to the population behaviour and state

$$u(t, x, c) = u(t, \theta, k) = -\alpha i - \omega_s s (k_s - 1)^2 - \omega_i i (k_i - 1)^2 \qquad \text{[S85]}$$
$$U_f(t) = -\alpha i_f \qquad \text{[S86]}$$

where the cleanup term can be derived analogously to Eq. C.2. The Hamiltonian is now given by

$$\begin{aligned} H &= u + \lambda \cdot F = u - \Delta R_0 k_s k_i s i - \lambda_i i \\ \Delta &= \lambda_s - \lambda_i \end{aligned} \qquad \text{[S87]}$$

The optimality condition ($\partial_k H = 0$) reduces to the following system of equations for $k^\star$

$$\begin{pmatrix} 2\omega_s s & \Delta R_0 s i \\ \Delta R_0 s i & 2\omega_i i \end{pmatrix} \cdot \begin{pmatrix} k_s^\star \\ k_i^\star \end{pmatrix} = 2 \begin{pmatrix} \omega_s s \\ \omega_i i \end{pmatrix} \quad . \qquad \text{[S88]}$$

which we can express as the following fixed-point problem

$$k^\star = \mathcal{C}(k^\star; t, \theta, \lambda) = \begin{pmatrix} 1 - \frac{R_0}{2\omega_s} i \Delta k_i^\star \\ 1 - \frac{R_0}{2\omega_i} s \Delta k_s^\star \end{pmatrix} \qquad \text{[S89]}$$

A closed form solution of these equations can be found as

$$\begin{pmatrix} k_s^\star \\ k_i^\star \end{pmatrix} = \frac{1}{d} \begin{pmatrix} 1 - \frac{R_0}{2\omega_s}\Delta_\theta i \\ 1 - \frac{R_0}{2\omega_i}\Delta_\theta s \end{pmatrix} \tag{S90}$$

$$d = 1 - \frac{R_0^2}{4\omega_s\omega_i}\Delta_\theta^2 si. \tag{S91}$$

Finally we give the equation and boundary condition for the costate dynamics ($\dot{\lambda} = -\partial_\theta H$). Only population costates exist in this formulation, but the costates of the individual would be defined analogously since they are a member of the population who is behaving according to k.

$$\begin{aligned} \dot{\lambda} &\equiv -\partial_\theta H(t, \theta, \lambda, k^\star) && \text{[S92]} \\ &= \begin{pmatrix} \omega_s(k_s^\star - 1)^2 + \Delta R_0 k_s^\star k_i^\star i \\ \alpha + \omega_i(k_i^\star - 1)^2 + \Delta R_0 k_s^\star k_i^\star s + \lambda_i \end{pmatrix} && \text{[S93]} \\ \dot{\theta} &\equiv F(t, \theta, k^\star) && \text{[S94]} \end{aligned}$$

with boundary conditions

$$\lambda(t_f) = \partial_\theta U_f(t_f, \theta(t_f)) = \begin{pmatrix} 0 \\ -\alpha \end{pmatrix}. \tag{S95}$$

Optimal behaviours that solve this optimal control problem are shown in Supplementary Text 5.B.

3. Altruistic Model

A. Motivation. The goal of the main paper is to present a model where people care about both themselves and the population. For this, we needed some sort of measure of altruism. The main paper includes a discussion of the ways that others have interpreted this, (6, 8, 10–14). We interpret altruism as the number of people in the population that an individual equates with themselves.

Define *Altruism* $\chi \geq 0$ to be the inverse of the number of population members that the individual would give equal value to themselves. For example, for altruism $\chi = 0.1$, an individual would equate their costs with the combined costs of 10 other people. This means for $\chi = 0$, the individual is completely selfish, as they would equate their costs with the costs of an infinite number of people. But for $\chi = 1$, the individual would value a random population member equally to themselves. One can think of this as modelling an ethic of reciprocity where everyone "Love[s] your neighbour as yourself". A whole population who acts like this would give the exact same effect as a theoretical 'social planner' would. Our model needs to reflect this.

B. Model Description.

B.1. Dynamics. One important observation is that if we assume that the individual can infect the population, then we cannot make the individual a part of the population, because the individual cannot infect themselves. Therefore this has transformed into a two player game between the individual and the population.

The individual (order 1) is interacting with a population (order $N - 1$). Consider the basic formulation of the SIR Dynamics

$$\begin{aligned} \frac{dS}{dt} &= -\beta \frac{I}{N} S \\ \frac{dI}{dt} &= \beta \frac{I}{N} S - \gamma I \\ \frac{dR}{dt} &= \gamma I \end{aligned} \tag{S96}$$

with $S + I + R = N$ the size of the population. We now want to split up the population into the 1 individual and the $N - 1$ 'rest of the population'. We incorporate behaviour and the individual's effect on the population as follows

$$\begin{aligned} \frac{dS}{dt} &= -\beta S \frac{1}{N}[k_i k_s I + \kappa_i k_s p_i] \\ \frac{dI}{dt} &= \beta S \frac{1}{N}[k_i k_s I + \kappa_i k_s p_i] - \gamma I \\ \frac{dR}{dt} &= \gamma I \end{aligned} \tag{S97}$$

where $S + I + R = N - 1$ and $p_i \in [0, 1]$ is the probability that the individual is infected. The denominator in the force of infection term is still N (instead of $N - 1$) because we can consider S, I, R to have size/order $N - 1$, so that the term inside

square brackets will have size/order N in total. We can rescale our parameters $\frac{S}{N-1}, \frac{I}{N-1}, \frac{R}{N-1} \to s, i, r$ and set $\epsilon = \frac{1}{N}$ to recover the θ population dynamics of Eq. 1.

$$\begin{aligned}
\dot{s} &= -R_0 k_s s((1-\epsilon)k_i i + \epsilon\kappa_i p_i) \\
\dot{i} &= R_0 k_s s((1-\epsilon)k_i i + \epsilon\kappa_i p_i) - i \\
\dot{p_s} &= -R_0 \kappa_s k_i p_s i \\
\dot{p_i} &= R_0 \kappa_s k_i p_s i - p_i
\end{aligned} \tag{S98}$$

Note that under a Nash equilibrium, the population dynamics reduces exactly to the individual dynamics for all values of ϵ.

In the sections below, we derive the corresponding optimality equations. Equilibrium dynamics are then found using the FBS Method presented in section 1.C.

B.2. Theory. Now we derive the optimality equations for altruistic individuals. Here, we consider that individuals care about themselves as well as the population. Futhermore, they are able to directly affect the population through their behavior κ (to order ϵ), but by definition they cannot directly control the behavior of the population. Thus, the state of the system is described by the individual behavior κ and state p, together with the population state θ. The population behavior k now plays the role of the external field ξ. To summarize, within the Optimal Control framework described above we have

$$x = (\theta, p) = ((s, i), (p_s, p_i)) \tag{S99}$$

$$c = \kappa = (\kappa_s, \kappa_i) \tag{S100}$$

$$\lambda = (\lambda_\theta, \lambda_p) = ((\lambda_s, \lambda_i), (\lambda_{p_s}, \lambda_{p_i})) \tag{S101}$$

$$\xi = k = (k_s, k_i) \tag{S102}$$

Where it is now necessary to introduce two sets of Lagrange multipliers $\lambda_p = (\lambda_{p_s}, \lambda_{p_i})$ and $\lambda_\theta = (\lambda_s, \lambda_i)$ to constrain the individual and population dynamics. For simplicity, we again define Δ to be the difference in the susceptible and individual costate values,

$$\Delta = (\Delta_\theta, \Delta_p) = (\lambda_s - \lambda_i, \lambda_{p_s} - \lambda_{p_i}) \tag{S103}$$

The costates for the population λ_s, λ_i represent the shadow cost that the individual associates with the general population being either susceptible or infected. We rescale these parameters to $\tilde{\lambda}$, such that the population costates can be interpreted as the shadow cost that the individual associates with an average population member being either susceptible or infected. For this, we define $\tilde{\lambda}$ as

$$\widetilde{\lambda} = \widetilde{J} \cdot \lambda \tag{S104}$$

$$\widetilde{\Delta} = \widetilde{J} \cdot \Delta \tag{S105}$$

$$\widetilde{J} = \begin{pmatrix} \frac{\epsilon}{1-\epsilon} & 0 \\ 0 & 1 \end{pmatrix} \tag{S106}$$

Note that the individual costates are unchanged, i.e., $\widetilde{\lambda}_p = \lambda_p$ and $\widetilde{\Delta}_p = \Delta_p$.

The constraint dynamics are

$$\begin{aligned}
F_\xi(t, x, c) = F_\xi(t, \theta, p, \kappa) &= \begin{pmatrix} F_\xi^\theta(t, \theta, p, \kappa) \\ F_\xi^p(t, \theta, p, \kappa) \end{pmatrix} \\
&= \begin{pmatrix} \begin{pmatrix} -R_0 k_s s\left((1-\epsilon)k_i i + \epsilon\kappa_i p_i\right) \\ R_0 k_s s\left((1-\epsilon)k_i i + \epsilon\kappa_i p_i\right) - i \end{pmatrix} \\ \begin{pmatrix} -R_0 \kappa_s \kappa_i p_s i \\ R_0 \kappa_s \kappa_i p_s i - p_i \end{pmatrix} \end{pmatrix}
\end{aligned} \tag{S107}$$

with a payoff function and cleanup term given by (Eq. 2 in the main paper)

$$\begin{aligned}
u_\xi(t, x, c) = u_k(t, \theta, p, \kappa) = &- \left(\alpha p_i + \omega_s p_s (\kappa_s - 1)^2 + \omega_i p_i (\kappa_i - 1)^2\right) \\
&- \chi(1/\epsilon - 1)\left(\alpha i + \omega_s s (k_s - 1)^2 + \omega_i i (k_i - 1)^2\right)
\end{aligned} \tag{S108}$$

$$U_f(t, \theta(t_f), p(t_f)) = -\alpha\left(p_i(t_f) + \chi(1/\epsilon - 1) i(t_f)\right) \tag{S109}$$

We have taken the same general form of the utility given by Eq. S58, except that now population level terms are introduced that will be effected by the individual, due to the dynamics presented in Eq. S98. Our idea of altruism is that the individual cares about others at a proportion χ compared to themselves. Hence, we take the selfish and utilitarian utilities in Eq. S64 and Eq. S85 and add them together, scaling the latter by $\chi \geq 0$. We have also assumed that infection and social distancing costs are the same for both the individual and the population (i.e., we use the same α, ω_s, and ω_i parameters for both). Now the individual cares about themselves and other population members, but these population level terms are affected by the individual

at order ϵ compared with their own utility terms. Hence, we also scale the population level terms by $(N-1) = (1/\epsilon - 1)$ to correct for this. Now, the utility represents how much the individual cares about the entire population, themselves included. As for the cleanup term U_f, one can make a similar argument to that expressed in section C.2 to say that this term should involve both i and p_i, both decaying exponentially for times larger than t_f. Thus, the cleanup term would be defined with the same scaling as the payoff. In the end, we will take the limit as $\epsilon \to 0$, i.e the population size goes to infinity. We are not concerning ourselves with quantifying exactly how much impact each individual has, and this knowledge will probably not be known to the individual either. Instead, we only want to model how the individual thinks of themselves in comparison to an average population member. Taking this limit allows us to do that. This preserves the 'mean field' status of our game (15).

The Hamiltonian for this system is

$$\begin{aligned} H_\xi(t,\theta,p,\kappa) &= u_\xi(t,\theta,p,\kappa) + \lambda \cdot F_\xi(t,\theta,p,\kappa) \\ &= -\left(\alpha p_i + \omega_s p_s(\kappa_s - 1)^2 + \omega_i p_i(\kappa_i - 1)^2\right) \\ &\quad - \chi(1/\epsilon - 1)\left(\alpha i + \omega_s s(k_s - 1)^2 + \omega_i i(k_i - 1)^2\right) \\ &\quad - \Delta_\theta R_0 k_s s((1-\epsilon)k_i i + \epsilon \kappa p_i) - \Delta_p R_0 \kappa_s p_s k_i i - \lambda_i i - \lambda_{p_i} p_i \end{aligned} \tag{S110}$$

We start by considering the optimality condition $\partial_\kappa H = 0$,

$$\begin{pmatrix} 2\omega_s p_s & 0 \\ 0 & 2\omega_i p_i \end{pmatrix} \cdot \begin{pmatrix} \kappa_s^\star \\ \kappa_i^\star \end{pmatrix} = 2\begin{pmatrix} \omega_s p_s - \frac{R_0}{2} i k_i p_s \widetilde{\Delta}_p \\ \omega_i p_i - \frac{R_0}{2} s k_s p_i (1-\epsilon)\widetilde{\Delta}_\theta \end{pmatrix} \tag{S111}$$

At the Nash equilibrium $(\theta = p, k = \kappa)$, in the infinite population limit $\epsilon \to 0$, we have

$$k^\star = k^\star(t,\theta,\lambda) = \lim_{\epsilon \to 0} \kappa_\xi^\star(t,\theta,p,\lambda)\Big|_{\theta=p,k=\kappa} \tag{S112}$$

$$= \mathcal{C}(k^\star; t,\theta,\lambda) = \begin{pmatrix} 1 - \frac{R_0}{2\omega_s} i k_i^\star \Delta_p \\ 1 - \frac{R_0}{2\omega_i} s k_s^\star \Delta_\theta \end{pmatrix} \tag{S113}$$

which provides the fixed-point definition used in our FBS update rule. The closed form solution for the optimal control is

$$k^\star = \frac{1}{d}\begin{pmatrix} 1 - \frac{R_0}{2\omega_s} i \widetilde{\Delta}_p \\ 1 - \frac{R_0}{2\omega_i} s \widetilde{\Delta}_\theta \end{pmatrix} \tag{S114}$$

$$d = 1 - \frac{R_0^2}{4\omega_s\omega_i} s i \widetilde{\Delta}_p \widetilde{\Delta}_\theta \tag{S115}$$

The costate dynamics are given by

$$\dot{\lambda}_\xi = -(\partial_{(\theta,p)} H_\xi)(t,\theta,p,\lambda,\kappa^\star) \tag{S116}$$

$$= \begin{pmatrix} \chi(1/\epsilon - 1)\omega_s(k_s - 1)^2 + R_0 k_s\left(k_i i(1-\epsilon) + \kappa_i^\star p_i \epsilon\right)\Delta_\theta \\ \chi(1/\epsilon - 1)\left(\alpha + \omega_i(k_i - 1)^2\right) + R_0 k_s k_i s(1-\epsilon)\Delta_\theta + R_0 \kappa_s^\star k_i p_s \Delta_p + \lambda_i \\ \omega_s(\kappa_s^\star - 1)^2 + R_0 \kappa_s^\star k_i i \Delta_p \\ \alpha + \omega_i(\kappa_i^\star - 1)^2 + R_0 k_s \kappa_i^\star s \epsilon \Delta_\theta + \lambda_{p_i} \end{pmatrix} \tag{S117}$$

$$\lambda_\xi(t_f) = \partial_{(\theta,p)} U_f(t_f, \theta(t_f), p(t_f)) \tag{S118}$$

$$= \begin{pmatrix} 0 \\ -\alpha\chi(1/\epsilon - 1) \\ 0 \\ -\alpha \end{pmatrix} \tag{S119}$$

from which we obtain the scaled costate dynamics to be $(\widetilde{\lambda} = \widetilde{J} \cdot \lambda)$

$$\dot{\widetilde{\lambda}}_\xi = \begin{pmatrix} \chi\omega_s(k_s - 1)^2 + R_0 k_s\left(k_i i(1-\epsilon) + \kappa_i^\star p_i \epsilon\right)\widetilde{\Delta}_\theta \\ \chi\left(\alpha + \omega_i(k_i - 1)^2\right) + R_0 k_s k_i s(1-\epsilon)\widetilde{\Delta}_\theta + \frac{\epsilon}{1-\epsilon} R_0 \kappa_s^\star k_i p_s \widetilde{\Delta}_p + \widetilde{\lambda}_i \\ \omega_s(\kappa_s^\star - 1)^2 + R_0 \kappa_s^\star k_i i \widetilde{\Delta}_p \\ \alpha + \omega_i(\kappa_i^\star - 1)^2 + R_0 k_s \kappa_i^\star s(1-\epsilon)\widetilde{\Delta}_\theta + \widetilde{\lambda}_{p_i} \end{pmatrix} \tag{S120}$$

$$\widetilde{\lambda}(t_f) = \widetilde{J} \cdot \lambda(t_f) = \begin{pmatrix} 0 \\ -\alpha(1-\epsilon)\chi \\ 0 \\ -\alpha \end{pmatrix} \tag{S121}$$

Finally, we consider the Nash solution in the $\epsilon \to 0$ limit,

$$\dot{\widetilde{\lambda}} = \lim_{\epsilon\to 0} \dot{\widetilde{\lambda}}_\xi \Big|_{\theta=p,k=\kappa} \tag{S122}$$

$$= \begin{pmatrix} \chi\omega_s(k_s^\star - 1)^2 + R_0 k_s^\star k_i^\star i\widetilde{\Delta}_\theta \\ \chi\left(\alpha + \omega_i(k_i^\star - 1)^2\right) + R_0 k_s^\star k_i^\star s\widetilde{\Delta}_\theta + \widetilde{\lambda}_i \\ \omega_s(k_s^\star - 1)^2 + R_0 k_s^\star k_i^\star i\widetilde{\Delta}_p \\ \alpha + \omega_i(k_i^\star - 1)^2 + R_0 k_s^\star k_i^\star s\widetilde{\Delta}_\theta + \widetilde{\lambda}_{p_i} \end{pmatrix} \tag{S123}$$

$$\widetilde{\lambda}(t_f) = \begin{pmatrix} 0 \\ -\alpha\chi \\ 0 \\ -\alpha \end{pmatrix} \tag{S124}$$

As expected, the corresponding state dynamics simply recover the SIR equations,

$$\dot{\theta} = F^\theta(t,\theta,k^\star) = \lim_{\epsilon\to 0} F_\xi^\theta(t,\theta,p,\kappa)\Big|_{\theta=p,k=\kappa} \tag{S125}$$

$$= F_\xi^p(t,\theta,p,\kappa^\star)\Big|_{\theta=p,k=\kappa} \tag{S126}$$

$$= \begin{pmatrix} -R_0 k_s^\star k_i^\star si \\ R_0 k_s^\star k_i^\star si - i \\ -R_0 k_s^\star k_i^\star si \\ R_0 k_s^\star k_i^\star si - i \end{pmatrix} \tag{S127}$$

B.3. Selfish Limit. The introduction of altruism χ allows us to scale between self-interested and utilitarian cases as presented in section 2.C and section 2.D. We will show in the following how the case $\chi = 0$ is equivalent to the purely self-interested case presented in section 2.C. When substituting $\chi = 0$, the equations for the population and individual costate dynamics can be effectively decoupled (see Eq. S123). The population costates are given by

$$\dot{\widetilde{\lambda}}_\theta = \begin{pmatrix} R_0 k_s^\star k_i^\star i\widetilde{\Delta}_\theta \\ R_0 k_s^\star k_i^\star s\widetilde{\Delta}_\theta + \widetilde{\lambda}_i \end{pmatrix} \qquad \widetilde{\lambda}_\theta(t_f) = \begin{pmatrix} 0 \\ 0 \end{pmatrix} \tag{S128}$$

Performing a variable substitution $(\widetilde{\lambda}_s, \widetilde{\lambda}_i) \to (\widetilde{\Delta}, \widetilde{\lambda}_i)$, we obtain

$$\dot{\widetilde{\Delta}}_\theta = -R_0 k_s^\star k_i^\star (s-i)\widetilde{\Delta}_\theta - \widetilde{\lambda}_i \tag{S129}$$

$$\dot{\widetilde{\lambda}}_i = R_0 k_s^\star k_i^\star s\widetilde{\Delta}_\theta + \widetilde{\lambda}_i \tag{S130}$$

$$\widetilde{\Delta}_\theta(t_f) = \widetilde{\lambda}_i(t_f) = 0 \tag{S131}$$

However, note that the end-time boundary conditions $\widetilde{\Delta}_\theta(t_f) = \widetilde{\lambda}_i(t_f) = 0$ are a fixed point of this system of equations. Therefore, we have $\widetilde{\Delta}_\theta(t) = \widetilde{\lambda}_i(t) = 0$, and $\widetilde{\lambda}_s(t) = 0$. Thus, the only non-trivial dynamics are those of the individual costates,

$$\dot{\widetilde{\lambda}}_p = \begin{pmatrix} \omega_s(k_s^\star - 1)^2 + R_0 k_s^\star k_i^\star i\widetilde{\Delta}_p \\ (\alpha + \omega_i(k_i^\star - 1)^2) + R_0 k_s^\star k_i^\star s\widetilde{\Delta}_\theta + \widetilde{\lambda}_{p_i} \end{pmatrix} \tag{S132}$$

$$= \begin{pmatrix} \omega_s(k_s^\star - 1)^2 + R_0 k_s^\star k_i^\star i\widetilde{\Delta}_p \\ (\alpha + \omega_i(k_i^\star - 1)^2) + \widetilde{\lambda}_{p_i} \end{pmatrix} \tag{S133}$$

$$\widetilde{\lambda}_p(t_f) = \begin{pmatrix} 0 \\ -\alpha \end{pmatrix} \tag{S134}$$

which exactly match those of the selfish model, Eq. S70, as required (recall that $\widetilde{\lambda}_p = \lambda_p$, $\widetilde{\Delta}_p = \Delta_p$). Likewise, the optimal behavior $k^\star$ is also that of the selfish model (Eq. S68)

$$k^\star = \frac{1}{d}\begin{pmatrix} 1 - \frac{R_0}{2\omega_s} i\widetilde{\Delta}_p \\ 1 - \frac{R_0}{2\omega_i} s\widetilde{\Delta}_\theta \end{pmatrix} \xrightarrow[\widetilde{\lambda}_\theta = 0]{} \begin{pmatrix} 1 - \frac{R_0}{2\omega_s} i\widetilde{\Delta}_p \\ 1 \end{pmatrix} \tag{S135}$$

B.4. Utilitarian Limit. We now consider the $\chi = 1$ limit of the altruistic model and show that it is equivalent to the utilitarian model in section 2.D. The population and individual costate dynamics are now given by

$$\dot{\widetilde{\lambda}}_\theta = \begin{pmatrix} \omega_s(k_s^\star - 1)^2 + R_0 k_s^\star k_i^\star i \widetilde{\Delta}_\theta \\ (\alpha + \omega_i(k_i^\star - 1)^2) + R_0 k_s^\star k_i^\star s \widetilde{\Delta}_\theta + \widetilde{\lambda}_i \end{pmatrix} \quad \text{[S136]}$$

$$\dot{\widetilde{\lambda}}_p = \begin{pmatrix} \omega_s(k_s^\star - 1)^2 + R_0 k_s^\star k_i^\star i \widetilde{\Delta}_p \\ (\alpha + \omega_i(k_i^\star - 1)^2) + R_0 k_s^\star k_i^\star s \widetilde{\Delta}_\theta + \widetilde{\lambda}_{p_i} \end{pmatrix} \quad \text{[S137]}$$

$$\widetilde{\lambda}_\theta(t_f) = \widetilde{\lambda}_p(t_f) = \begin{pmatrix} 0 \\ -\alpha \end{pmatrix} \quad \text{[S138]}$$

Here these two sets of equations do not decouple, as the dynamics for $\widetilde{\lambda}_p$ are slaved to those of $\widetilde{\lambda}_\theta$ due to the $\widetilde{\Delta}_\theta$ dependence in the equation for $\dot{\widetilde{\lambda}}_{p_i}$. However, note that the dynamical equations for $\widetilde{\lambda}_i$ and $\widetilde{\lambda}_{p_i}$ are indentical, and that the later is independent of $\widetilde{\lambda}_{p_s}$. Furthermore, since the boundary conditions are also equal, $\widetilde{\lambda}_i(t_f) = \widetilde{\lambda}_{p_i}(t_f) = -\alpha$, the solutions for the infected costates should also coincide, i.e., $\widetilde{\lambda}_i(t) = \widetilde{\lambda}_{p_i}(t)$. The remaining equations for the susceptible costates are then seen to have the same functional form, with matching boundary conditions,

$$\dot{\widetilde{\lambda}}_s = \omega_s(k_s^\star - 1)^2 + R_0 k_s^\star k_i^\star i(\widetilde{\lambda}_s - \widetilde{\lambda}_i) = \mathcal{F}(\widetilde{\lambda}_s, \widetilde{\lambda}_i = \widetilde{\lambda}_{p_i}) \quad \text{[S139]}$$

$$\dot{\widetilde{\lambda}}_{p_s} = \omega_s(k_s^\star - 1)^2 + R_0 k_s^\star k_i^\star i(\widetilde{\lambda}_{p_s} - \widetilde{\lambda}_{p_i}) = \mathcal{F}(\widetilde{\lambda}_{p_s}, \widetilde{\lambda}_{p_i} = \widetilde{\lambda}_i) \quad \text{[S140]}$$

such that $\widetilde{\lambda}_\theta(t) = \widetilde{\lambda}_p(t) = \widetilde{\Delta}(t)$, with the costate dynamics equivalent to that of the Utilitarian model, Eq. S92. The resulting optimal control $k^\star$ is

$$k^\star = \frac{1}{d}\begin{pmatrix} 1 - \frac{R_0}{2\omega_s} i\widetilde{\Delta} \\ 1 - \frac{R_0}{2\omega_i} s\widetilde{\Delta} \end{pmatrix} \quad \text{[S141]}$$

$$d = 1 - \frac{R_0^2}{4\omega_s\omega_i} si\widetilde{\Delta}^2 \quad \text{[S142]}$$

which is exactly the Utilitarian optimal control of Eq. S88.

C. Analytic estimates for behaviour. In what follows, we calculate the analytic solution for the behaviour and epidemic dynamics assuming time-independent behaviour. This is a reasonable assumption for the Indefinite Suppression Nash equilibrium. Then, we calculate the variation of the utility for time-independent behaviour and derive analytic estimates for lines in (χ, i_0) parameter space along which the value of the rational time-independent behaviour remains constant.

C.1. Analytic solution for constant behaviour. We observe that the numerical solution of the control problem exhibits a transition between two Nash equilibria: one leading to herd immunity and one in which the disease is suppressed until the vaccination time t_f. We also notice that on the suppression side of the transition, $k_s = \kappa_s \approx 1$ and $k_i = \kappa_i$ are all approximately constant in time. For constant behaviour, the analytic solution of the SIR equations is known (5, 16, 17). We restate here the SIR equations studied in this work (see Eq. 1) but also include the dynamics of the recovered compartment

$$\begin{aligned} \dot{s} &= -R_0 k_s s((1-\epsilon)k_i i + \epsilon\kappa_i p_i) \\ \dot{i} &= R_0 k_s s((1-\epsilon)k_i i + \epsilon\kappa_i p_i) - i \\ \dot{r} &= i \\ \dot{p_s} &= -R_0 \kappa_s k_i p_s i \\ \dot{p_i} &= R_0 \kappa_s k_i p_s i - p_i \\ \dot{p_r} &= p_i \end{aligned} \quad \text{[S143]}$$

with $i(0) = p_i(0) = i_0$, $s(0) = p_s(0) = s_0 = 1 - i_0$, and $r(0) = p_r(0) = 0$ as boundary conditions. Here, we use the notation $\epsilon = 1/N$. Even though we are only interested in the Nash equilibrium for which the individual dynamics and the collective dynamics end up being identical, we keep the distinction between the two until we explicitly calculate the Nash equilibrium. Recalling that $\dot{r} = i$ and $\dot{p_r} = p_i$, the recovered fraction r and individual probability p_r can be used to parametrize the progress of the disease instead of time

$$\begin{aligned} r(t) &= \int_0^t i dt \\ p_r(t) &= \int_0^t p_i dt \end{aligned} \quad \text{[S144]}$$

Then we can write down the solution directly

$$
\begin{aligned}
s(r,p_r) &= s_0 \exp\left[-R_0 k_s \left((1-\epsilon)k_i r + \epsilon \kappa_i p_r\right)\right] \\
i(r,p_r) &= 1 - s(r,p_r) - r \\
p_s(r,p_r) &= s_0 \exp\left[-R_0 \kappa_s k_i r\right] \\
p_i(r,p_r) &= 1 - p_s(r,p_r) - p_r
\end{aligned} \quad \text{[S145]}
$$

The final state of the epidemic is given by $r(t \to \infty) = r_\infty$ and $p_r(t \to \infty) = p_{r_\infty}$. We can derive a self-consistency condition for the end state by inserting $i(t) \to 0$ and $p_i(t) \to 0$ for $t \to \infty$ into

$$
\begin{aligned}
r_\infty &= 1 - s_\infty = 1 - s_0 \exp\left[-R_0 k_s \left((1-\epsilon)k_i r_\infty + \epsilon \kappa_i p_{r_\infty}\right)\right] \\
p_{r_\infty} &= 1 - p_{s_\infty} = 1 - s_0 \exp\left[-R_0 \kappa_s k_i r_\infty\right]
\end{aligned} \quad \text{[S146]}
$$

At the Nash equilibrium, we know that $p_{r_\infty} = r_\infty$, etc. Then, with the definition

$$
\hat{R}_0 = R_0 k_s [(1-\epsilon)k_i + \epsilon \kappa_i] \quad \text{[S147]}
$$

we can write the self-consistency condition on the end state concisely as

$$
r_\infty = 1 - s_0 \exp\left[-\hat{R}_0 r_\infty\right] \quad \text{[S148]}
$$

This expression has the formal solution

$$
r_\infty = 1 + W(-s_0 \hat{R}_0 \exp[-\hat{R}_0])/\hat{R}_0 \quad \text{[S149]}
$$

with product logarithm W, also known as the Lambert W function (18).

Since $i = 1 - r - s = 1 - r - s_0 \exp[-\hat{R}_0 r]$ we can numerically reconstruct time with

$$
t = \int_0^r \frac{dr'}{1 - r' - s_0 \exp[-\hat{R}_0 r']}. \quad \text{[S150]}
$$

Having obtained the analytic solution for the dynamics given any constant behaviour, we can calculate the utility, calculate the Nash equilibrium self-consistently, and perform the infinite system size limit ($\epsilon \to 0$), as follows.

C.2. Nash equilibrium. We restate the utility as defined in Eq. 2

$$
\begin{aligned}
U =& \int_0^{t_f} u\, dt + U_f \\
u =& -\chi(N-1)\left(\alpha i + \omega_s s (k_s - 1)^2 + \omega_i i (k_i - 1)^2\right) \\
& - \left(\alpha p_i + \omega_s p_s (\kappa_s - 1)^2 + \omega_i p_i (\kappa_i - 1)^2\right) \\
U_f =& -\alpha p_i(t_f) - \chi(N-1)\alpha i(t_f)
\end{aligned} \quad \text{[S151]}
$$

We make the simplifying assumption that $\omega_s = \omega_i = \omega$. From here on, we will again work with $\epsilon = 1/N$. The population utility scales with $N - 1 = 1 - 1/\epsilon$ but since we are ultimately interested in the $\epsilon \to 0$ limit, it is sufficient (in that it leaves the limit unchanged) and slightly more compact in notation to write the population scaling as $1/\epsilon$.

Since we assume that $k_i = \kappa_i$ be constant in time, calculating its Nash equilibrium value does not require variational analysis anymore. It is self-consistently given by the κ_i for which

$$
0 = \lim_{\varepsilon \to 0} \frac{\partial}{\partial \kappa_i} U \quad \text{[S152]}
$$

Since we assume that $k_s = \kappa_s \approx 1$, we can drop the social distancing terms for the susceptible compartment and can write more concisely

$$
\begin{aligned}
0 =& \lim_{\varepsilon \to 0} \frac{\partial}{\partial \kappa_i} \int_0^{t_f} \left[-\alpha p_i - \frac{\alpha\chi}{\epsilon} i - \omega p_i (\kappa_i - 1)^2 - \frac{\omega\chi}{\epsilon} i (k_i - 1)^2\right] dt \\
&+ \lim_{\varepsilon \to 0} \frac{\partial}{\partial \kappa_i} [-\alpha p_i(t_f) - \frac{\alpha\chi}{\epsilon} i(t_f)]
\end{aligned} \quad \text{[S153]}
$$

Using the analytic rescaling of time via r and p_r, we rewrite the integrals as

$$
\begin{aligned}
0 =& \lim_{\varepsilon \to 0} \frac{\partial}{\partial \kappa_i} \int_0^{p_{rf}} \left[-\alpha - \omega(\kappa_i - 1)^2\right] dp_r \\
&+ \lim_{\varepsilon \to 0} \frac{\partial}{\partial \kappa_i} \int_0^{r_f} \left[-\frac{\alpha\chi}{\epsilon} - \frac{\omega\chi}{\epsilon}(k_i - 1)^2\right] dr \\
&+ \lim_{\varepsilon \to 0} \frac{\partial}{\partial \kappa_i} [-\alpha p_i(t_f) - \frac{\alpha\chi}{\epsilon} i(t_f)]
\end{aligned} \quad \text{[S154]}
$$

Integrating and flipping the sign yields

$$0 = \lim_{\varepsilon\to 0} \frac{\partial}{\partial \kappa_i} \left[\left(\alpha + \omega(\kappa_i - 1)^2\right) p_{r_f} + \left(\alpha + \omega(k_i - 1)^2\right) \frac{\chi}{\epsilon} r_f + \alpha p_{i_f} + \frac{\alpha\chi}{\epsilon} i_f \right] \quad \text{[S155]}$$

Since $\partial p_{r_f}/\partial\kappa_i \sim \epsilon$ and $\partial p_{i_f}/\partial\kappa_i \sim \epsilon$, these terms will vanish in the limit $\epsilon \to 0$ and we find

$$\begin{aligned} 0 = {} & 2\omega(\kappa_i - 1)\left[\lim_{\varepsilon\to 0} p_{r_f}\right] + \left(\alpha + \omega(k_i - 1)^2\right)\chi \left[\lim_{\varepsilon\to 0} \frac{1}{\epsilon}\frac{\partial r_f}{\partial \kappa_i}\right] \\ & + \alpha\chi \left[\lim_{\varepsilon\to 0} \frac{1}{\epsilon}\frac{\partial i_f}{\partial \kappa_i}\right] \end{aligned} \quad \text{[S156]}$$

For $\alpha = 0$, the equation has the trivial solution $k_i = \kappa_i = 1$. For $\alpha > 0$, in order to proceed, we need to calculate the expressions in brackets. However, for the other terms, we can already assume a Nash equilibrium, $k_i = \kappa_i \neq 1$. Then, provided we know how to evaluate the brackets, we can assume constant k_i and solve for χ to find lines of compassion values in (χ, i_0) parameter space along which the equilibrium behaviour would be constant

$$\chi = \frac{-2\omega(k_i - 1)\left[\lim_{\varepsilon\to 0} p_{r_f}\right]}{(\alpha + \omega(k_i - 1)^2)\left[\lim_{\varepsilon\to 0} \frac{1}{\epsilon}\frac{\partial r_f}{\partial \kappa_i}\right] + \alpha \left[\lim_{\varepsilon\to 0} \frac{1}{\epsilon}\frac{\partial i_f}{\partial \kappa_i}\right]} \quad \text{[S157]}$$

In what follows, we evaluate this expression for special cases.

Infinite vaccination time We assume that the course of the epidemic is such that at t_f the epidemic has reached a situation for which $i(t_f) \approx i_\infty = 0$, e.g. for $t_f \to \infty$. Then we can use Eq. S149 to yield with $\lim_{\varepsilon\to 0} \hat{R}_0 = R_0 k_i$ and $s_0 = 1 - i_0$

$$\begin{aligned} \hat{W} &= W[\exp(-k_i R_0)(-1 + i_0)k_i R_0] \\ \lim_{\varepsilon\to 0}\left[p_{r_f}\right] &= \lim_{\varepsilon\to 0}[r_\infty] = 1 + \hat{W}/(R_0 k_i) \\ \lim_{\varepsilon\to 0}\left[\frac{1}{\epsilon}\frac{\partial r_f}{\partial \kappa_i}\right] &= \lim_{\varepsilon\to 0}\left[\frac{1}{\epsilon}\frac{\partial r_\infty}{\partial \kappa_i}\right] = -\frac{\hat{W}\cdot(k_i R_0 + \hat{W})}{k_i^2 R_0(1 + \hat{W})} \\ \lim_{\varepsilon\to 0}\left[\frac{1}{\epsilon}\frac{\partial i_f}{\partial \kappa_i}\right] &= 0 \end{aligned} \quad \text{[S158]}$$

We insert these expressions into Eq. S157 and obtain

$$\chi = \frac{2\omega k_i(k_i - 1)(1 + \hat{W})}{(\alpha + \omega(k_i - 1)^2)\,\hat{W}} \quad \text{[S159]}$$

The line for which the equilibrium is given assuming $k_i = \kappa_i = 0$, i.e. perfect suppression of the disease, and inserting this into Eq. S159

$$\chi_0 = \frac{2\omega}{(1 - i_0)R_0(\alpha + \omega)} \quad \text{[S160]}$$

This equation is shown in Fig. 1B and Fig. 3 as the red line. To the right of this line, $\chi > \chi_0$, Eq. S159 predicts an equilibrium with $k_i = \kappa_i < 0$, but the bounds on the control still enforce $k_i = \kappa_i = 0$.

The line for which the equilibrium is given by $k_i = \kappa_i = 1/(s_0 R_0)$ with $s_0 = 1 - i_0$, i.e. the infectious use the largest possible constant behaviour k_i for which it is guaranteed that $di/dt < 0$ for all times, can be expressed to leading order for small i_0 as

$$\chi_\infty = \frac{12\omega(R_0 - 1)\sqrt{i_0}}{(3\sqrt{2} - 5\sqrt{i_0})(\omega - 2R_0\omega + R_0^2(\alpha + \omega))} \quad \text{[S161]}$$

This equation is shown in Eq. 1a as the pink line.

Transition line between suppression and herd immunity for finite t_f Here we don't assume that $i_f \approx 0$ and that therefore r_f can be approximated by r_∞. Instead we make use of Eq. S150 to calculate an explicit expression for $t_f(r_f)$ and invert it to yield $r_f(t_f)$. In practice, we cannot analytically evaluate Eq. S150, but must make an asymptotic expansion of the integrand in r around 0 up to second order

$$t_f \approx \int_0^{r_f} \frac{dr'}{i_0 + r'(-1 + (1 - i_0)\hat{R}_0) - r'^2(1 - i_0)\hat{R}_0^2/2} \quad \text{[S162]}$$

The integrand has poles r_1 and r_2

$$r_{1,2} = \frac{(1 - i_0)\hat{R}_0 - 1 \pm \sqrt{1 - 2(1 - i_0)\hat{R}_0 + (1 - i_0^2)\hat{R}_0^2}}{(1 - i_0)\hat{R}_0^2} \quad \text{[S163]}$$

with the naming convention such that $r_2 < 0 \le r < r_1$. Integration then yields

$$t_f \approx \frac{2}{(1-i0)\hat{R}_0^2}\ \frac{1}{r_1 - r_2} \ln\left[\frac{(r_f - r_2)r_1}{(r_f - r_1)r_2}\right]$$
$$= \frac{2}{(1-i0)\hat{R}_0^2}\ \frac{1}{r_1 - r_2}\left(\ln\left[\frac{r_f - r_2}{r_1 - r_f}\right] + \ln\left[\frac{-r_1}{r_2}\right]\right) \quad \text{[S164]}$$

Our approach involves a further approximation. We neglect the term $\ln(-r_1/r_2)$. The value of this term vanishes when $\hat{R}_0 = 1/(1-i_0)$, corresponding to the behaviour $k_i = 1/(s_0 R_0)$. However, in what follows we also need to evaluate the derivative $\partial r_f/\partial \kappa_i$. We choose to also neglect this term when computing the derivative. Although this approximation appears somewhat unprincipled, it works remarkably well, see Fig. 3A. We therefore proceed with the following form for t_f,

$$t_f \approx \frac{2}{(1-i0)\hat{R}_0^2}\ \frac{1}{r_1 - r_2} \ln\left[\frac{r_f - r_2}{r_1 - r_f}\right] \quad \text{[S165]}$$

This can then be inverted to yield

$$r_f = \frac{r_1 \exp[(r_1 - r_2)\hat{R}_0^2(1-i_0)t_f/2] + r_2}{1 + \exp[(r_1 - r_2)\hat{R}_0^2(1-i_0)t_f/2]} \quad \text{[S166]}$$

This expression can be used to evaluate the terms in brackets in Eq. S156. Explicitly, we calculate

$$\lim_{\varepsilon \to 0}\left[\frac{1}{\epsilon}\frac{\partial i_f}{\partial \kappa_i}\right] = \lim_{\varepsilon \to 0}\left[\frac{1}{\epsilon}\frac{\partial r_f}{\partial \kappa_i}\right](-1 + (1-i_0)R_0 k_i \exp[-R_0 k_i r_f])$$
$$+ (1-i_0)R_0 r_f \exp[-R_0 k_i r_f]; \quad \text{[S167]}$$

Then, we expand Eq. S156 up to leading order in small i_0, and solve for χ

$$\chi_{t_f} = \frac{2 i_0 (R_0 - 1) t_f \omega}{R_0^2 \alpha + \omega(R_0 - 1)^2} \quad \text{[S168]}$$

The lines described by Eq. S161 and Eq. S168 meet at a crossover value

$$i_0^* = \frac{9 - 30/t_f - 3\sqrt{9 - 60/t_f}}{25} \sim \frac{2}{t_f^2} \quad \text{for } t_f \text{ large} \quad \text{[S169]}$$

Since this scales as $i_0^* \approx 2/t_f^2$, the corresponding crossover altruism parameter scales as

$$\chi^* \approx \frac{4\omega(R_0 - 1)}{(R_0^2\alpha + \omega(R_0 - 1)^2)t_f} \quad \text{[S170]}$$

D. Creating the Heatmaps. In order to obtain the utility and control heatmaps in Fig. 1 and Fig. 3, we run a full FBS Method (see section 1.C) for each value of altruism χ and i_0. However, as is shown Fig. 1A, the same coordinate can converge to one of two Nash equilibria.

The FBS Method requires an initial guess for the control k, from which it is able to find a converged control representing a Nash equilibria. In order to find herd immunity solutions, for each value of i_0 we start with an initial guess of $k_s(t) = k_i(t) = 1$ to obtain the equilibria for the smallest value of altruism $\chi = 1 \times 10^{-6}$. Then for each subsequent value of χ increasing, we use the converged control of the previous value of χ as the initial control guess. This finds herd immunity solutions until some large χ where indefinite suppression becomes the only rational solution. To find all Indefinite Suppression solutions, we start with $k_s(t) = 1, k_i(t) = 0$ for the largest value of altruism $\chi = 1$. Then for each subsequent value of χ decreasing, we again use the converged control of the previous value of χ as the initial control guess. This technique is able to find more rational solutions near the edge of the indefinite suppression branch than merely using an initial control guess of $k_s(t) = 1, k_i(t) = 0$ for all χ.

We never found any other Nash equilibria except for the two described above, but cannot exclude the possibility that there might be additional equilibria.

4. Altruistic Asymptomatic Model

One assumption in our current model is the idea that individuals know exactly when they become infected and can start infecting others. This is reflected by a transition from being susceptible to being infected and instantly changing ones behaviour from κ_s to κ_i. This then also allows a full suppression solution to the problem to exist, as the infected individuals can completely suppress the disease through their social distancing.

For epidemics such as the SARS-CoV-2 epidemic, massive asymptomatic populations were infecting others, despite not knowing they had the disease since they were showing no symptoms (19). This motivates the consideration of behavioural, asymptomatic disease models. Ones which consider a compartment of infected individuals who are acting as if they are susceptible, because they don't know that they are infected.

A. Model Description. A description of the model dynamics and utility function can be found in the Methods and Materials section 0.B.

A.1. Asymptomatic Cleanup Term under perfect vaccination. At the end-time t_f, we again make the assumption that a 'perfect vaccine' becomes available which instantly immunises all susceptible individuals at the end-time $p_s(t > t_f) = s(t > t_f) = 0$. In this asymptomatic case, we assume that the vaccine does not stop an already asymptomatic individual from going on to show symptoms. At the end-time, the remaining asymptomatic population either recover or show symptoms and enter the infected class, however no new asymptomatics enter the compartment.

The disease dynamics are reduced to the equations

$$\begin{aligned} \dot{a} &= -(\gamma_{\mathrm{AR}} + \gamma_{\mathrm{AI}})a \\ \dot{i} &= \gamma_{\mathrm{AI}}a - \gamma_{\mathrm{IR}}i \\ \dot{p}_a &= -(\gamma_{\mathrm{AR}} + \gamma_{\mathrm{AI}})p_a \\ \dot{p}_i &= \gamma_{\mathrm{AI}}p_a - \gamma_{\mathrm{IR}}p_i \end{aligned} \tag{S171}$$

The asymptomatic equations can be solved directly after time t_f as

$$\begin{aligned} a(t > t_f) &= a_f \exp[-(\gamma_{\mathrm{AR}} + \gamma_{\mathrm{AI}})(t - t_f)] \\ p_a(t > t_f) &= p_{a,f} \exp[-(\gamma_{\mathrm{AR}} + \gamma_{\mathrm{AI}})(t - t_f)]. \end{aligned} \tag{S172}$$

where $a_f = a(t_f), p_{a,f} = p_i(a_f)$. Substituting these into the infected equations after time t_f and solving then gives

$$\begin{aligned} i(t > t_f) &= -a_f A \exp[-(\gamma_{\mathrm{AR}} + \gamma_{\mathrm{AI}})(t - t_f)] + (i_f + a_f A) \exp[-\gamma_{\mathrm{IR}}(t - t_f)] \\ p_i(t > t_f) &= -p_{a,f} A \exp[-(\gamma_{\mathrm{AR}} + \gamma_{\mathrm{AI}})(t - t_f)] + (p_{i,f} + p_{a,f} A) \exp[-\gamma_{\mathrm{IR}}(t - t_f)] \end{aligned} \tag{S173}$$

where $i_f = i(t_f), p_{i,f} = p_i(i_f)$ and

$$A = \frac{\gamma_{\mathrm{AI}}}{\gamma_{\mathrm{AI}} + \gamma_{\mathrm{AR}} - \gamma_{\mathrm{IR}}}. \tag{S174}$$

Since there is no risk to becoming infected, and no risk of passing the infection to others, there is no reason to socially distance. So $k(t > t_f) = \kappa(t > t_f) = (1, 1)$. By taking the utility as defined in Eq. 9 and substituting Eq. S171, we solve for the late time utility U_f to obtain

$$U_f = \alpha \left(\frac{p_{a,f} A}{\gamma_{\mathrm{AI}} + \gamma_{\mathrm{AR}}} - \frac{p_{i,f} + p_{a,f} A}{\gamma_{\mathrm{IR}}} \right) + \chi\alpha(1/\epsilon - 1) \left(\frac{a_f A}{\gamma_{\mathrm{AI}} + \gamma_{\mathrm{AR}}} - \frac{i_f + a_f A}{\gamma_{\mathrm{IR}}} \right). \tag{S175}$$

A.2. Theory. We can then formulate and solve an optimal control problem as described in section 1.B. Similar to the fully symptomatic case, we remove the dynamics for one of the state variables since this is a closed system where $s + a + i + r_a + r_i = 1, p_s + p_a + p_i + p_{r_a} + p_{r_i} = 1$. We remove r_i, p_{r_i} due to the behaviour of this class having no affect on the system dynamics or utility.

Under the generalised Optimal Control framework, we have

$$x = (\theta, p) = ((s, a, i, r_a), (p_s, p_a, p_i, p_{r_a})) \tag{S176}$$

$$c = \kappa = (\kappa_s, \kappa_i) \tag{S177}$$

$$\lambda = (\lambda_\theta, \lambda_p) = ((\lambda_s, \lambda_a, \lambda_i, \lambda_{r_a}), (\lambda_{p_s}, \lambda_{p_a}, \lambda_{p_i}, \lambda_{p_{r_a}})) \tag{S178}$$

$$\xi = k = (k_s, k_i) \tag{S179}$$

Where we still introduce two sets of Lagrange multipliers $\lambda_p = (\lambda_{p_s}, \lambda_{p_i})$ and $\lambda_\theta = (\lambda_s, \lambda_i)$ to constrain the individual and population dynamics. For simplicity, we again define Δ to be the difference in the susceptible and asymptomatic costate values,

$$\Delta = (\Delta_\theta, \Delta_p) = (\lambda_s - \lambda_a, \lambda_{p_s} - \lambda_{p_a}) \tag{S180}$$

As before, population costates are rescaled to $\tilde{\lambda}$, defined as

$$\widetilde{\lambda} = \widetilde{J} \cdot \lambda \tag{S181}$$

$$\widetilde{\Delta} = \widetilde{J} \cdot \Delta \tag{S182}$$

$$\widetilde{J} = \begin{pmatrix} \frac{\epsilon}{1-\epsilon} & 0 \\ 0 & 1 \end{pmatrix} \tag{S183}$$

The constraint dynamics are

$$F_\xi(t,x,c) = F_k(t,\theta,p,\kappa) \tag{S184}$$

$$= \begin{pmatrix} F_k^\theta(t,\theta,p,\kappa) \\ F_k^p(t,\theta,p,\kappa) \end{pmatrix} \tag{S185}$$

$$= \begin{pmatrix} \begin{pmatrix} -\beta_a s k_s((1-\epsilon)ak_s + \epsilon p_a\kappa_s) - \beta_i s k_s((1-\epsilon)ik_i + \epsilon p_i\kappa_i) \\ \beta_a s k_s((1-\epsilon)ak_s + \epsilon p_a\kappa_s) + \beta_i s k_s((1-\epsilon)ik_i + \epsilon p_i\kappa_i) - (\gamma_{\mathrm{AR}} + \gamma_{\mathrm{AI}})a \\ \gamma_{\mathrm{AI}}a - \gamma_{\mathrm{IR}}i \\ \gamma_{\mathrm{AR}}a \end{pmatrix} \\ \begin{pmatrix} -\beta_a p_s\kappa_s a k_s - \beta_i p_s\kappa_s i k_i \\ \beta_a p_s\kappa_s a k_s + \beta_i p_s\kappa_s i k_i - (\gamma_{\mathrm{AR}} + \gamma_{\mathrm{AI}})p_a \\ \gamma_{\mathrm{AI}}p_a - \gamma_{\mathrm{IR}}p_i \\ \gamma_{\mathrm{AR}}p_a \end{pmatrix} \end{pmatrix} \tag{S186}$$

with a payoff function and cleanup term given by

$$u_\xi(t,x,c) = u_k(t,\theta,p,\kappa) = -\left(\alpha p_i + \omega_s(p_s + p_a + p_{r_a})(\kappa_s - 1)^2 + \omega_i p_i(\kappa_i - 1)^2\right) \tag{S187}$$

$$- \chi(\frac{1}{\epsilon} - 1)\left(\alpha i + \omega_s(s + a + r_a)(k_s - 1)^2 + \omega_i i(k_i - 1)^2\right) \tag{S188}$$

$$U_f(t,\theta(t_f),p(t_f)) = \alpha\left(\frac{p_{a,f}A}{\gamma_{\mathrm{AI}} + \gamma_{\mathrm{AR}}} - \frac{p_{i,f} + p_{a,f}A}{\gamma_{\mathrm{IR}}}\right) \tag{S189}$$

$$+ \chi\alpha(1/\epsilon - 1)\left(\frac{a_f A}{\gamma_{\mathrm{AI}} + \gamma_{\mathrm{AR}}} - \frac{i_f + a_f A}{\gamma_{\mathrm{IR}}}\right) \tag{S190}$$

For brevity, we skip the intermediate steps as done in section B.2. The final costate dynamics are

$$\dot{\widetilde{\lambda}} = \begin{pmatrix} \omega_s\chi(k_s^\star - 1)^2 + k_s^\star\widetilde{\Delta}_\theta(\beta_a a k_s^\star + \beta_i i k_i^\star) \\ \omega_s\chi(k_s^\star - 1)^2 + \widetilde{\Delta}_\theta\beta_a s k_s^{\star 2} + (\gamma_{\mathrm{AR}} + \gamma_{\mathrm{AI}})\widetilde{\lambda}_a - \gamma_{\mathrm{AI}}\widetilde{\lambda}_i - \gamma_{\mathrm{AR}}\widetilde{\lambda}_{r_a} \\ \chi(\alpha + \omega_i(k_i^\star - 1)^2) + \beta_i\widetilde{\Delta}_\theta s k_s^\star k_i^\star + \gamma_{\mathrm{IR}}\widetilde{\lambda}_i \\ \omega_s\chi(k_s^\star - 1)^2 \\ \omega_s(k_s^\star - 1)^2 + k_s^\star\widetilde{\Delta}_p(\beta_a a k_s^\star + \beta_i i k_i^\star) \\ \omega_s(k_s^\star - 1)^2 + \widetilde{\Delta}_\theta\beta_a s k_s^{\star 2} + \gamma_{\mathrm{AR}}(\widetilde{\lambda}_{p_a} - \widetilde{\lambda}_{p_{r_a}}) + \gamma_{\mathrm{AI}}(\widetilde{\lambda}_{p_a} - \widetilde{\lambda}_{p_i}) \\ \alpha + \omega_i(k_i^\star - 1)^2 + \beta_i\widetilde{\Delta}_\theta s k_s^\star k_i^\star + \gamma_{\mathrm{IR}}\widetilde{\lambda}_{p_i} \\ \omega_s(k_s^\star - 1)^2 \end{pmatrix} \tag{S191}$$

$$\widetilde{\lambda}(t_f) = \begin{pmatrix} 0 \\ \chi\alpha A\left(\frac{1}{\gamma_{\mathrm{AR}} + \gamma_{\mathrm{AI}}} - \frac{1}{\gamma_{\mathrm{IR}}}\right) \\ -\frac{\chi\alpha}{\gamma_{\mathrm{IR}}} \\ 0 \\ 0 \\ A\left(\frac{1}{\gamma_{\mathrm{AR}} + \gamma_{\mathrm{AI}}} - \frac{1}{\gamma_{\mathrm{IR}}}\right) \\ -\frac{\alpha}{\gamma_{\mathrm{IR}}} \\ 0 \end{pmatrix} \tag{S192}$$

The fixed-point update rule for the FBS method is now given by

$$k^\star = \mathcal{C}(k^\star; t,\theta,\lambda) = \begin{pmatrix} 1 - \frac{\beta_a s a k_s^\star(\widetilde{\Delta}_\theta + \widetilde{\Delta}_p) + \beta_i s i k_i^\star\widetilde{\Delta}_p}{2\omega_s(s + a + r_a)} \\ 1 - \frac{\beta_i k_s^\star s\widetilde{\Delta}_\theta}{2\omega_i} \end{pmatrix}. \tag{S193}$$

B. Calculating the Basic Reproduction Number. Calculating R_0 is now much more complicated than in the standard SIR model. Here we use a next generation matrix approach to calculate the basic reproduction number (20–22).The method goes as follows. We define the next generation matrix as $K = -T\Sigma^{-1}$ where T is the transmission matrix and Σ is the transition matrix. The entries k_{ij} of this matrix are then the average number of compartment i cases generated by a compartment j case in a fully susceptible population. The basic reproduction number R_0 is then given by the largest eigenvalue of K. Whilst there are five compartments in our disease model, we only need to consider compartments a and i since all the other compartments are connected to one of these two. These matrices are given by

$$T = \begin{pmatrix} \beta_a s & \beta_i s \\ 0 & 0 \end{pmatrix} \quad \text{[S194]}$$

for which we consider a fully susceptible population ($s = 1$), then

$$\Sigma = \begin{pmatrix} -(\gamma_{\mathrm{AR}} + \gamma_{\mathrm{AI}}) & 0 \\ \gamma_{\mathrm{AI}} & -\gamma_{\mathrm{IR}} \end{pmatrix}. \quad \text{[S195]}$$

So finally

$$K = -T\Sigma^{-1} = \frac{1}{\gamma_{\mathrm{IR}}(\gamma_{\mathrm{AR}} + \gamma_{\mathrm{AI}})} \begin{pmatrix} \beta_a \gamma_{\mathrm{IR}} + \beta_i \gamma_{\mathrm{AI}} & \beta_i(\gamma_{\mathrm{AR}} + \gamma_{\mathrm{AI}}) \\ 0 & 0 \end{pmatrix}. \quad \text{[S196]}$$

The largest eigenvalue of which is then

$$R_0 = \frac{\beta_a \gamma_{\mathrm{IR}} + \beta_i \gamma_{\mathrm{AI}}}{\gamma_{\mathrm{IR}}(\gamma_{\mathrm{AR}} + \gamma_{\mathrm{AI}})}. \quad \text{[S197]}$$

In order to fully compare results between the asymptomatic and fully symptomatic cases, we need to ensure that this value of R_0 has the same time scaling as our previous results. For this, we first make the modelling assumption that $\beta = \beta_a = \beta_i$, so that infections to susceptibles from both asymptomatics and infected individuals happen at the same rate. In the fully symptomatic model (See section 2.A), we then simply set the timescale $\gamma = 1$, such that then $R_0 = \beta$. We adopt a similar approach here where we want to pick the transition rates such that

$$\frac{\gamma_{\mathrm{IR}} + \gamma_{\mathrm{AI}}}{\gamma_{\mathrm{IR}}(\gamma_{\mathrm{AR}} + \gamma_{\mathrm{AI}})} = 1. \quad \text{[S198]}$$

We observe that if we set $\gamma_{\mathrm{IR}} = \gamma_{\mathrm{AR}} = 1$, then we are allowed to vary $\gamma_{\mathrm{AI}} > 0$ to be any value whilst keeping the same time scaling. This still gives us the freedom to tune other aspects of the disease whilst keeping R_0 fixed. We choose to vary the proportion of infections which go on to show symptoms, given by

$$\sigma = \frac{\gamma_{\mathrm{AI}}}{1 + \gamma_{\mathrm{AI}}}. \quad \text{[S199]}$$

Using σ, we observe that the fully symptomatic case should be derived in the limit $\sigma \to 1$, corresponding to $\gamma_{\mathrm{AI}} \to \infty$. Effectively, individuals move so quickly out of the asymptomatics class and into the infected class that they just appear to show symptoms as soon as they are infected.

Using the values of $\beta = R_0$ and $\gamma_{\mathrm{IR}} = \gamma_{\mathrm{AR}} = 1$, as well as defining $\gamma_{\mathrm{AI}} = \frac{\sigma}{1-\sigma}$ we restate all the necessary equations for the optimal control problem for clarity. The utility function remains the same as Eq. S188 but we can rewrite the cleanup term from Eq. S189 as

$$U_f(t, \theta(t_f), p(t_f)) = -\alpha \left(\sigma p_{a,f} + p_{i,f}\right) - \chi\alpha(1/\epsilon - 1)\left(\sigma a_f + i_f\right). \quad \text{[S200]}$$

The system dynamics from Eq. S186 become

$$F_k(t, \theta, p, \kappa) = \begin{pmatrix} \begin{pmatrix} -R_0 s k_s \left((1-\epsilon) a k_s + \epsilon p_a \kappa_s + (1-\epsilon) i k_i + \epsilon p_i \kappa_i\right) \\ R_0 s k_s \left((1-\epsilon) a k_s + \epsilon p_a \kappa_s + (1-\epsilon) i k_i + \epsilon p_i \kappa_i\right) - \left(\frac{1}{1-\sigma}\right) a \\ \left(\frac{\sigma}{1-\sigma}\right) a - i \\ a \end{pmatrix} \\ \begin{pmatrix} -R_0 p_s \kappa_s \left(a k_s + i k_i\right) \\ R_0 p_s \kappa_s \left(a k_s + i k_i\right) - \left(\frac{1}{1-\sigma}\right) p_a \\ \left(\frac{\sigma}{1-\sigma}\right) p_a - p_i \\ p_a \end{pmatrix} \end{pmatrix} \quad \text{[S201]}$$

The costate equations from Eqs. S191- S192 become

$$\dot{\widetilde{\lambda}} = \begin{pmatrix} \omega_s \chi (k_s^\star - 1)^2 + R_0 k_s^\star \widetilde{\Delta}_\theta (a k_s^\star + i k_i^\star) \\ \omega_s \chi (k_s^\star - 1)^2 + R_0 \widetilde{\Delta}_\theta s k_s^{\star 2} + (\widetilde{\lambda}_a - \widetilde{\lambda}_{r_a}) + \left(\frac{\sigma}{1-\sigma}\right)(\widetilde{\lambda}_a - \widetilde{\lambda}_i) \\ \chi(\alpha + \omega_i (k_i^\star - 1)^2) + R_0 \widetilde{\Delta}_\theta s k_s^\star k_i^\star + \widetilde{\lambda}_i \\ \omega_s \chi (k_s^\star - 1)^2 \\ \omega_s (k_s^\star - 1)^2 + R_0 k_s^\star \widetilde{\Delta}_p (a k_s^\star + i k_i^\star) \\ \omega_s (k_s^\star - 1)^2 + R_0 \widetilde{\Delta}_\theta s k_s^{\star 2} + (\widetilde{\lambda}_{p_a} - \widetilde{\lambda}_{p_{r_a}}) + \left(\frac{\sigma}{1-\sigma}\right)(\widetilde{\lambda}_{p_a} - \widetilde{\lambda}_{p_i}) \\ \alpha + \omega_i (k_i^\star - 1)^2 + R_0 \widetilde{\Delta}_\theta s k_s^\star k_i^\star + \widetilde{\lambda}_{p_i} \\ \omega_s (k_s^\star - 1)^2 \end{pmatrix} \quad \text{[S202]}$$

$$\widetilde{\lambda}(t_f) = \begin{pmatrix} 0 \\ -\chi\alpha\sigma \\ -\chi\alpha \\ 0 \\ 0 \\ -\alpha\sigma \\ -\alpha \\ 0 \end{pmatrix} \quad \text{[S203]}$$

Finally, the fixed-point solution to the optimal control given by Eq. S193 is now

$$k^\star = \mathcal{C}(k^\star; t, \theta, \lambda) = \begin{pmatrix} 1 - \frac{R_0 s a k_s^\star (\widetilde{\Delta}_\theta + \Delta_p) + R_0 s i k_i^\star \widetilde{\Delta}_p}{2\omega_s (s + a + r_a)} \\ 1 - \frac{R_0 k_s^\star s \widetilde{\Delta}_\theta}{2\omega_i} \end{pmatrix}. \quad \text{[S204]}$$

C. Obtaining χ_c. The value of altruism for which an Indefinite Suppression solution starts to exist is vital in our work. Specifically, for the asymptomatic heatmap in Fig. 4A. Finding this value required different section for regions above and below the black line given by Eq. 13 in that figure.

For regions above the black line, where infected self-isolation leads to indefinite suppression, we used a similar technique as finding the indefinite suppression solutions above, and we characterised χ_c as the χ that had the largest jump in the average value of k_i when χ was decreased to the next value. But there were some convergence issues for values of χ approaching χ_c (from above). We wanted to ensure these points converged to be certain we had found χ_c. The problem was that some oscillations had propagated through k_i that wouldn't reduce in amplitude. To resolve this, if after 200000 iterations of the FBS a point had not converged, we would replace the first n time-points in k_i and replace them all with their average value. This initial run was done with convergence parameters $\nu = 1 \times 10^{-3}, \delta = 1 \times 10^{-6}$. We then ran the algorithm for another 150000 iterations with $\nu = 1 \times 10^{-5}, \delta = 1 \times 10^{-8}$ using this new control as the initial guess. If it did not converge within this time, we repeated this process until it did. The specific value of n we used varied depending on the values of R_0, σ.

For regions below the black line, some of the indefinite suppression solutions did not quite converge. This was due to large positive and negative swings in the early time costate values throughout the FBS method. This lead to large control updates and would leave large spikes in the value of k_i at early times. The value of altruism before the drop onto the Herd Immunity branch always converged (with parameters $\nu = 1 \times 10^{-3}, \delta = 1 \times 10^{-6}$), this is what we call the critical altruism χ_c. However, to solve this non-convergence issue for other altruism values, we removed the bounds on the control (which was previously set to $0 < \kappa_s, \kappa_i < 2$). This allowed the control to learn the true unbounded value instead of being clipped above and below at these values. However, it is important to apply these bounds to the control when using it in each iterations forward calculation of the state, backwards calculation of the costate and the update rule. This is because negative controls are unphysical, and so the state, costate and new control guess must be evaluated with the bounded controls. It is only when we save the value of the control (after mixing) and check for convergence where this is done on the unbounded control.

Finally, for the heatmap in Fig. 4A the critical altruism was found by starting from a value of $\chi = 10$ and using each converged control as the initial guess for the next smallest value of control. However, in Fig. 4B another run was done starting from the smallest altruism of $\chi = 1 \times 10^{-6}$ and moving to large altruism. This allowed us to find multiple Nash equilibria where they existed.

5. Supporting Results

A. Self Interested Results. Using the optimality equations derived in section 2.C we run a FBS (see section 1.C) to solve for the optimal behaviours that satisfy the Nash equilibrium. We give three different example solutions in Fig. S1, obtained by varying the cost of infection $\alpha = 5, 10, 20$. In all cases, we obtain a herd immunity solution, where the disease is allowed to pass through the population, seeing a peak of infection, and then dying out as the number of people left to infect decreases. This occurs because the minority infected population perform no social distancing throughout the course of the epidemic. After all, a once infected a selfish individual has no reason to incur the cost of social distancing. This means that the majority susceptible population is forced to social distance in order to reduce their likelihood of being infected. For a disease with a

higher cost of infection, we see that rational susceptible individuals will social distance more, start that distancing earlier, and also take much longer to return to the standard population behaviour at $\kappa_s = 1$.

The costate variables show how the perceived cost of being in each compartment changes over time. The shadow cost of being infected is constant (See Fig. S1f), since the only cost incurred is that of being infected oneself. But the shadow cost of being susceptible is more complex (See Fig. S1c). The shadow cost starts off large (large negative) because there is a high potential to eventually become infected. And once the social distancing starts, this shadow cost decreases as this potential becomes lower. By remaining susceptible as other individuals are infected, the potential to become infected decreases as herd immunity is approached. However, unlike the infected individuals, social distancing is still performed by a susceptible individual, and so the cost remains high. Eventually, once the epidemic is over and the infected proportion is becoming exponentially smaller (i.e Herd Immunity is reached), the susceptibles do not social distance any more and their probability of being infected is very low. Hence why the shadow cost of being susceptible is near zero.

B. Utilitarian Results. Using the optimality equations derived in section 2.D we run a FBS (see section 1.C) to obtain the population behaviours which lead to the highest possible utility value. We give three different examples of converged solution in Fig. S2, varying the cost of infection each time $\alpha = 5, 0.5, 0.05$. In this scenario, since the infected individuals know that they are infected and care about the costs of the population, they completely self isolate. This reduces the rate of spread of the disease to 0 and the epidemic simply dies out exponentially (as can be seen in Fig. S2d). We call this type of epidemic '*Indefinite Suppression*'. Note that this occurs even for very small costs of infection.

C. Coexistence of Both Equilibria. As stated in the main paper, both equilibria exist over much of the parameter space. The difference between solutions is very pronounced for small i_0 and χ, but a second region appears to emerge for large i_0, see Fig. 1B.

We zoom in to this region of high i_0 and χ in Fig S4, which shows the difference between the utility values of Herd Immunity equilibria $\hat{U}_{HI}$ and Indefinite Suppression equilibria $\hat{U}_{IS}$. The Indefinite Suppression branch still shows incredible alignment with χ_∞ (see pink dashed line), pardoning a few blue pixels above this estimate following the edge of the Herd Immunity branch. However, on this finer scale it is clear that these points are a continuation of the main coexistence region in the bottom left.

The region of coexistence in the top right is qualitatively distinct. Here, the exact definitions between Herd Immunity and Indefinite Suppression becomes blurred. Infected individuals self-isolating leads to suppression, but for such large values of i_0 Herd Immunity cannot be avoided. For values $i_0 \gtrsim 1.26 \times 10^{-1}$, it becomes a rational strategy for the susceptible sub-population to self isolate whilst the infected sub-population perform no social distancing, i.e. $k_s = 0, k_i = 1$. After a small period of time, the behaviours swap and return to $k_s = 1, k_i = 0$, leading to a bang-bang control strategy. As i_0 increases, the period of time that the susceptibles maintain their social distancing before this swap occurs also increases. Such equilibria are traced out by $\hat{U}_{HI}$ and provides the smooth transition between Herd Immunity and Indefinite Suppression type solutions. Yet, for the region enclosed by our estimate for χ_0, it remains a rational strategy for solely the infected sub-population to self-isolate, $k_s = 1, k_i = 0$. Such equilibria are traced out by $\hat{U}_{IS}$.

Both equilibria result in the same epidemic state; no infection occurs. But since the behaviours required to achieve these equilibria differ, they result in different utility values. In the vast majority of cases, the infected sub-population self-isolating is the better strategy. The exception is only for very large i_0 and χ values, see red region in Fig S4. This region of coexistence has its border exactly along our estimate for χ_0, due to it only being possible for no disease transmission.

D. Varying Parameters. Throughout the main paper, we took the fixed parameters of $\alpha = 5$, $\omega = 1$, $R_0 = 3$, $t_f = 100$. Changing these parameters have various effects on the existence of Nash equilibria.

In Fig. S5a and b, the effect of changing the cost of infection α relative to the cost of social distancing ω is shown, where Fig. S5a shows a cost of infection of $\alpha = 0.5$ and Fig. S5b shows $\alpha = 50$. If the individuals in a population do not consider it as bad to be infected compared to social distancing, then it takes a higher level of altruism in order to achieve an indefinite suppression solution and for the infected population to reduce their behaviour to $k_i = 0$. But if the disease is more severe with a higher cost of infection, then it takes a smaller level of altruism for an indefinite suppression strategy to be possible, and for the infected population to rationally self-isolate. The values of α, ω in the utility function are not immediately obvious from given disease data. Deriving these values from given disease and behavioural data is left for future work. However, with this model we are able to see the general trends of increasing and decreasing the costs of infection by orders of magnitude.

In Fig. S5c and d, the effect of changing the basic reproduction number R_0 of the disease is shown, where Fig. S5c shows a disease with $R_0 = 2$ and Fig. S5b shows $R_0 = 4$. The affect on the critical altruism for the R_0 values shown here is less extreme than the affect on changing α shown is, but a similar response is observed. For a disease with a smaller R_0, it takes more altruism for the population to target indefinite suppression. The disease is spreading at a slower rate, and thus less social distancing is required from the susceptibles This reduces the overall utility cost significantly. This means that it takes more altruism for the infected individuals to start social distancing because the benefit to their utility in doing this is smaller than in the case with a larger R_0. In this case, much more severe social distancing is put in place by the susceptibles and the overall number of people infected is larger. This results in a lower utility, thus the utility gained by infected social distancing is larger and so it takes a lower level of altruism to achieve this result. See Fig. S6 for details on the dynamics of these three diseases.

Finally, in Fig. S5e and f, the affect of changing the end-time of the epidemic t_f is shown, where Fig. S5e shows an end-time of $t_f = 50$ and Fig. S5f shows $t_f = 300$. This end time corresponds to a perfect vaccine becoming available, where all the

susceptible individuals instantly become immune to the disease and the remaining infected individuals recover over time. This shows the accuracy of the $t_f \to \infty$ prediction, as the critical altruism χ_c is in line with this estimate for the larger $t_f = 300$ value. The Indefinite Suppression points to the left of this asymptote occur because individuals are behaving in a way that suppresses the disease specifically to the end time. So if that end time of the epidemic is larger, then the altruism needed for infected individuals to target a behaviour of $k_i = \frac{1}{R_0}$ is going to be larger (by observing Eq. 5).

Whilst not shown in the utility plots in Fig. S5, the boundary estimate for the toe region given by χ_{t_f} in Eq. S168 is still an accurate estimate for the contour where $\min(k_i)= \frac{1}{R_0}$.

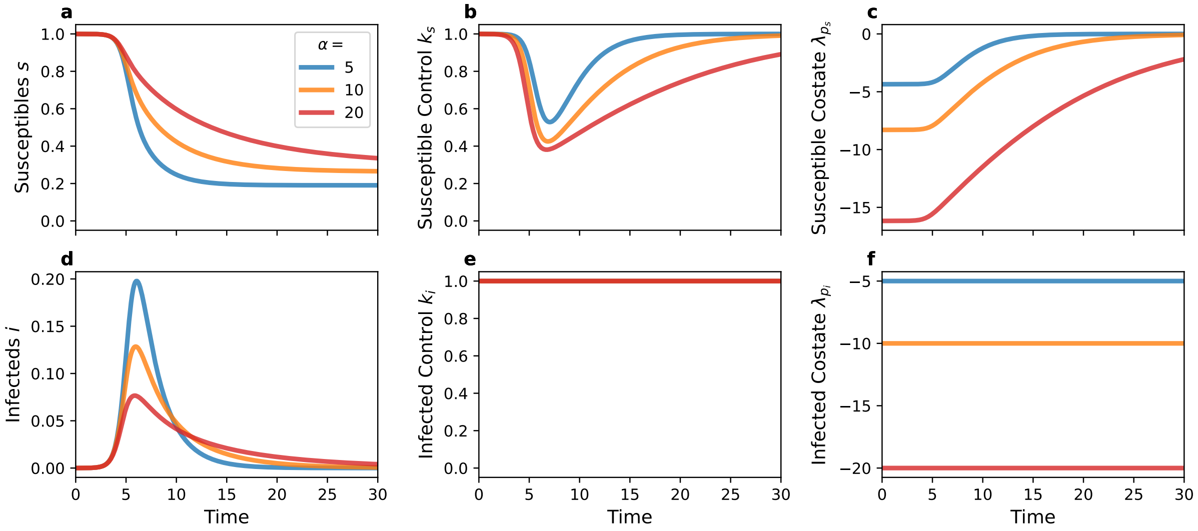

Fig. S1. Purely Self Interested Behaviour. Plots of the behaviour of a purely self interested population in the presence of three different epidemics. Each epidemic has values $\omega_s = \omega_i = 1, R_0 = 3, i_0 = 10^{-5}, t_f = 100$ and what differentiates them is the perceived cost of infected $\alpha = 5$ (red), $\alpha = 10$ (orange), $\alpha = 20$ (blue). (A) the susceptible fraction, their behaviour (B), and their costate variable representing 'shadow costs' associated with being in that compartment (C) are shown as functions of time. (D) the infected fraction, their behaviour (E), and their costate variable (F) are shown as functions of time.

 Mark P. Lynch, Simon K. Schnyder, John J. Molina, Ryoichi Yamamoto, Matthew S. Turner

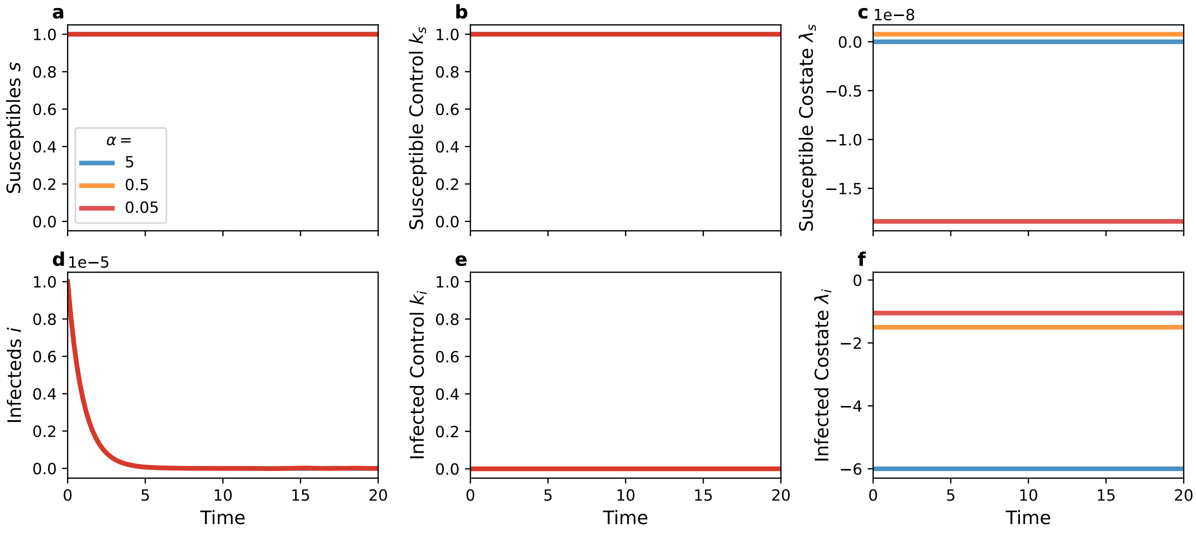

Fig. S2. Purely Utilitarian Behaviour. Plots of the behaviour of a population seeking the global maximum of utility given three different proportions of initial infected individuals. Each epidemic has values $i_0 = 10^{-5}, \omega_s = \omega_i = 1, R_0 = 3, t_f = 100$ and what differentiates them is the cost of infection $\alpha = 5$ (blue), $\alpha = 0.5$ (orange), $\alpha = 0.05$ (red). The state of the epidemic is shown in panels (A) the proportion of susceptibles; and (B) the proportion infected (shown with a logarithmic scale). The self-interested behaviours are shown in panels (B) the behaviour of the susceptibles; and (e) the behaviour of the infected. The costate variables representing 'shadow costs' associated with being in each subclass are given in panels (C) costate for susceptibles; and (f) costate for infected.

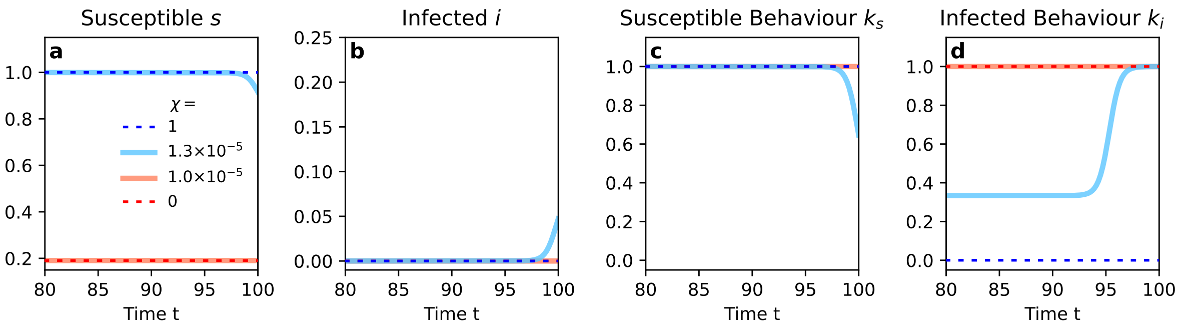

Fig. S3. Late-time dynamics. The late-time dynamics of the susceptible (a) and the infectious compartments (b), as well as the corresponding behaviours (c,d) are shown for initial infection level $i_0 = 10^{-5}$ for representative values of altruism χ on either side of the boundary of the Indefinite Suppression equilibrium at $\chi = \chi_c$, see corresponding coloured points in Fig. 1. Here we show results for $\alpha = 5$, $R_0 = 3$, $t_f = 100$. Same as Fig. 2, but now focusing on the range $80 < t < t_f = 100$.

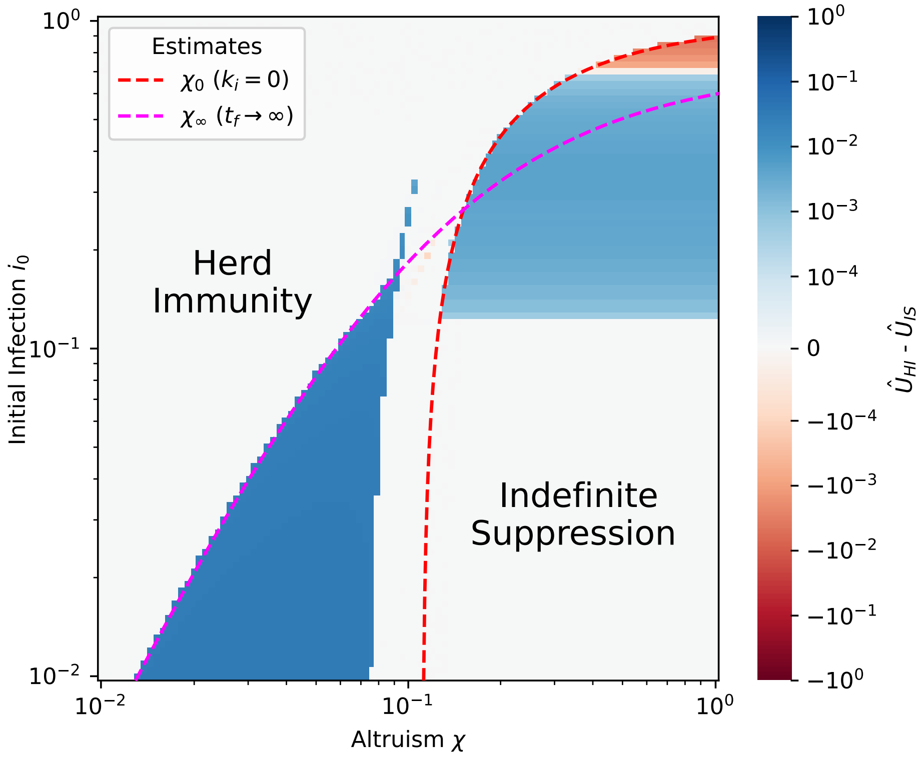

Fig. S4. Multiple equilibria exist for large i_0. The difference in scaled utility value between the Herd Immunity utility $\hat{U}_{HI}$ and the Indefinite Suppression utility $\hat{U}_{IS}$. Utility values are shown on a logorithmic scale from $1 \to 10^{-4}$ and $-10^{-4} \to -1$ and on a linear scale between $10^{-4} \to 10^{-4}$. Blue regions indicate where Indefinite Suppression is better than Herd Immunity, whereas red regions show the inverse. The analytical estimates for χ_0 (red dashed line) and χ_∞ (magenta dashed line) are also shown (see Eqs.S160 and S161).

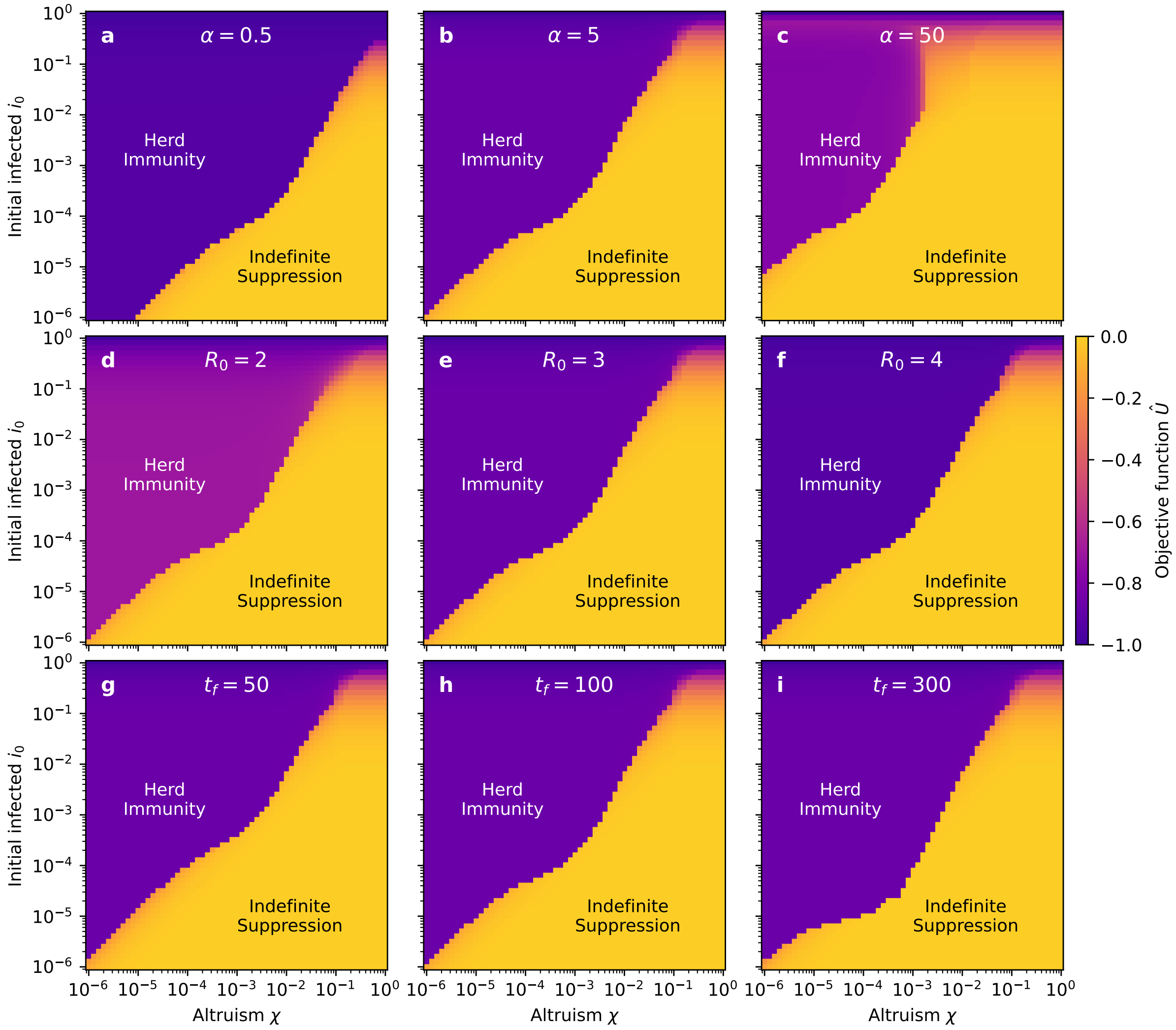

Fig. S5. The utility landscape varies quantitatively with the parameters, but not qualitatively. The highest value of $\hat{U}$ is shown for each altruism χ and initial infected fraction i_0. The base variables are $\alpha = 5, \omega = 1, R_0 = 3, t_f = 100$, the data for which is shown in the centre column of panels. This data was also presented in Fig. 1b. In the left and right columns of panels the results of a change in one of these variables as indicated in the panel are shown.

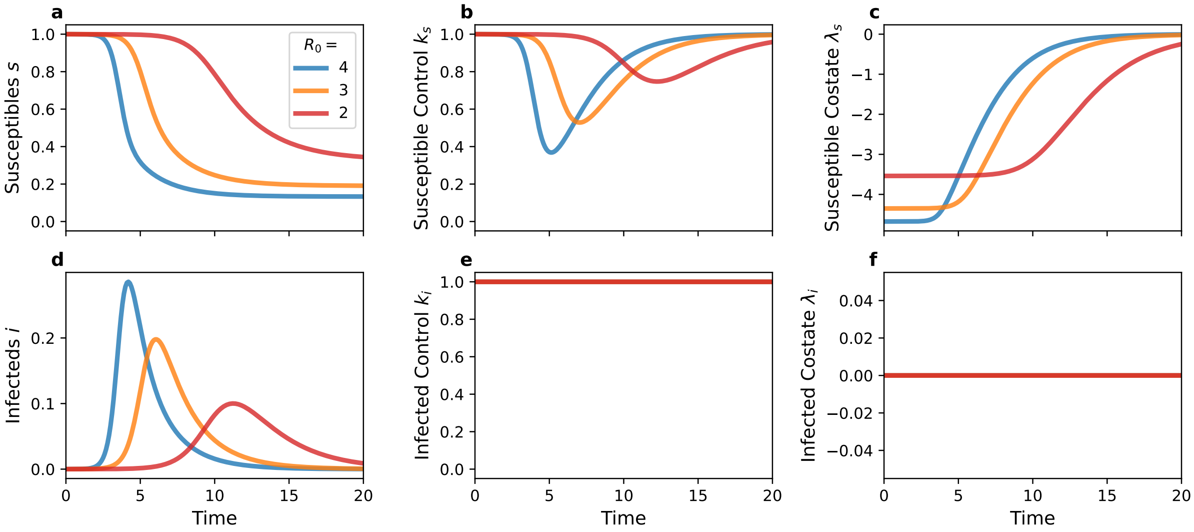

Fig. S6. Different Diseases in Purely Self Interested Populations. Nash Equilibrium Disease Dynamics of three different diseases spreading throughout perfectly self interested populations. The diseases are characterised by their basic reproductions numbers $R_0 = 2$ (red), $R_0 = 3$ (orange), $R_0 = 4$ (blue). Here, the rates of recovery are all the same with a time unit of 1.

References

1. IM Gelfand, SV Fomin, *Calculus of Variations.* (Dover Publications), (2000).
2. GJ Sussman, J Wisdom, *Structure and Interpretation of Classical Mechanics.* (MIT Press), 2 edition, (2014).
3. BC Chachuat, Nonlinear and dynamic optimization: From theory to practice, (Automatic Control Laboratory, EPFL, Switzerland), Technical report (2007).
4. S Lenhart, JT Workman, *Optimal control applied to biological models.* (Chapman and Hall/CRC), (2007).
5. WO Kermack, AG McKendrick, A contribution to the mathematical theory of epidemics. *Proc. Royal Soc. London. Ser. A, Containing Pap. a Math. Phys. Character* **115**, 700–721 (1927).
6. ZA Bethune, A Korinek, Covid-19 infection externalities: Trading off lives vs. livelihoods, (National Bureau of Economic Research), Working Paper 27009 (2020).
7. SK Schnyder, JJ Molina, R Yamamoto, MS Turner, Rational social distancing policy during epidemics with limited healthcare capacity. *PLoS Comput. Biol.* **19**, e1011533 (2023).
8. M Farboodi, G Jarosch, R Shimer, Internal and external effects of social distancing in a pandemic. *J. Econ. Theory* **196**, 105293 (2021).
9. M Makris, F Toxvaerd, Great expectations: Social distancing in anticipation of pharmaceutical innovations. *Covid Econ.* **56** (2020).
10. S Bosi, C Camacho, D Desmarchelier, Optimal lockdown in altruistic economies. *J. Math. Econ.* **93**, 102488 (2021).
11. MF Quaas, et al., The social cost of contacts: Theory and evidence for the first wave of the covid-19 pandemic in germany. *Plos one* **16**, e0248288 (2021).
12. LM Brotherhood, P Kircher, C Santos, M Tertilt, , et al., An economic model of the covid-19 pandemic with young and old agents: Behavior, testing and policies, (CESifo), Working Paper w202014 (2021).
13. L Alfaro, E Faia, N Lamersdorf, F Saidi, Altruism, social interactions, and the course of a pandemic. *Eur. Econ. Rev.* **161**, 104625 (2024).
14. F Toxvaerd, Contacts, altruism and competing externalities. *Eur. Econ. Rev.* **167**, 104794 (2024).
15. M Nourian, PE Caines, epsilon-nash mean field game theory for nonlinear stochastic dynamical systems with major and minor agents. *SIAM J. on Control. Optim.* **51**, 3302–3331 (2013).
16. JC Miller, A Note on the Derivation of Epidemic Final Sizes. *Bull. Math. Biol.* **74**, 2125–2141 (2012).
17. T Harko, FS Lobo, MK Mak, Exact analytical solutions of the Susceptible-Infected-Recovered (SIR) epidemic model and of the SIR model with equal death and birth rates. *Appl. Math. Comput.* **236**, 184–194 (2014).
18. RM Corless, GH Gonnet, DEG Hare, DJ Jeffrey, DE Knuth, On the Lambert W Function. *Adv. Comput. Math.* **5**, 329–359 (1996).
19. DP Oran, EJ Topol, Prevalence of asymptomatic sars-cov-2 infection: a narrative review. *Annals internal medicine* **173**, 362–367 (2020).
20. O Diekmann, J Heesterbeek, MG Roberts, The construction of next-generation matrices for compartmental epidemic models. *J. royal society interface* **7**, 873–885 (2010).
21. O Diekmann, JAP Heesterbeek, *Mathematical epidemiology of infectious diseases: model building, analysis and interpretation.* (John Wiley & Sons) Vol. 5, (2000).
22. P Van den Driessche, J Watmough, Reproduction numbers and sub-threshold endemic equilibria for compartmental models of disease transmission. *Math. biosciences* **180**, 29–48 (2002).
